# The Potential Role of Fisetin, a Flavonoid in Cancer Prevention and Treatment

**DOI:** 10.3390/molecules27249009

**Published:** 2022-12-17

**Authors:** Arshad Husain Rahmani, Ahmad Almatroudi, Khaled S. Allemailem, Amjad Ali Khan, Saleh A. Almatroodi

**Affiliations:** 1 Department of Medical Laboratories, College of Applied Medical Sciences, Qassim University, Buraydah 51542, Saudi Arabia; 2 Department of Basic Health Sciences, College of Applied Medical Sciences, Qassim University, Buraydah 51542, Saudi Arabia

**Keywords:** fisetin, cancer, cell signaling pathways, synergistic effect, bioavailability

## Abstract

Cancer is a main culprit and the second-leading cause of death worldwide. The current mode of treatment strategies including surgery with chemotherapy and radiation therapy may be effective, but cancer is still considered a major cause of death. Plant-derived products or their purified bioactive compounds have confirmed health-promoting effects as well as cancer-preventive effects. Among these products, flavonoids belong to polyphenols, chiefly found in fruits, vegetables and in various seeds/flowers. It has been considered to be an effective antioxidant, anti-inflammatory and to play a vital role in diseases management. Besides these activities, flavonoids have been revealed to possess anticancer potential through the modulation of various cell signaling molecules. In this regard, fisetin, a naturally occurring flavonoid, has a confirmed role in disease management through antioxidant, neuro-protective, anti-diabetic, hepato-protective and reno-protective potential. As well, its cancer-preventive effects have been confirmed via modulating various cell signaling pathways including inflammation, apoptosis, angiogenesis, growth factor, transcription factor and other cell signaling pathways. This review presents an overview of the anti-cancer potential of fisetin in different types of cancer through the modulation of cell signaling pathways based on in vivo and in vitro studies. A synergistic effect with anticancer drugs and strategies to improve the bioavailability are described. More clinical trials need to be performed to explore the anti-cancer potential and mechanism-of-action of fisetin and its optimum therapeutic dose.

## 1. Introduction

Cancer is a main culprit regarding health problems worldwide, which is a group of diseases caused by the abnormal growth of cells with invasive potential [1]. This disease is the principal cause of deaths worldwide, with approximately 10 million deaths and a projected 19.3 million cases in 2020, and this is predicted to reach 28.4 million new cases in 2040, a rise of 47% [2]. In spite of widespread global awareness, as well as the development of multi-targeted therapeutic opportunities, the mortality rates from cancer are still quite high [3].

The current modes of treatment including surgery, chemotherapy and radiation therapy may be effective but also cause adverse effects on health such as fatigue, hair loss, constipation, fever, vomiting and mouth sores. However, approaches with inexpensive strategies with fewer side effects are of prime interest for health science researchers to manage the cancer. In this vista, natural products have confirmed their role in cancer prevention and treatment through the modulation of cell signaling pathways.

Plants are fascinating sources of innovative molecules due to their confirmed efficacy, adequate bioavailability and easy applicability in human consumption, and plant products have become a source of modern medicine [4,5]. Because of the rich source of natural products with low side effects, they are becoming more vital than synthetic medication [6].

Flavonoids possess numerous biological effects including anti-cancer activities [7]. Previous studies have reported that some natural products or their purified compounds including thymoquinone [8], green tea [9], curcumin [10], baicalein [11], quercetin [12], berberine [13], apigenin [14] and ginger [15] play a significant role in cancer management. These compounds play a role in cancer protection through the modulation of various cell signaling pathways including inflammation, antioxidant potential, tumor suppression gene, apoptosis, angiogenesis, growth factor, signal transducer, activator of transcription 3 (STAT3) and other pathways.

**Figure 1:**
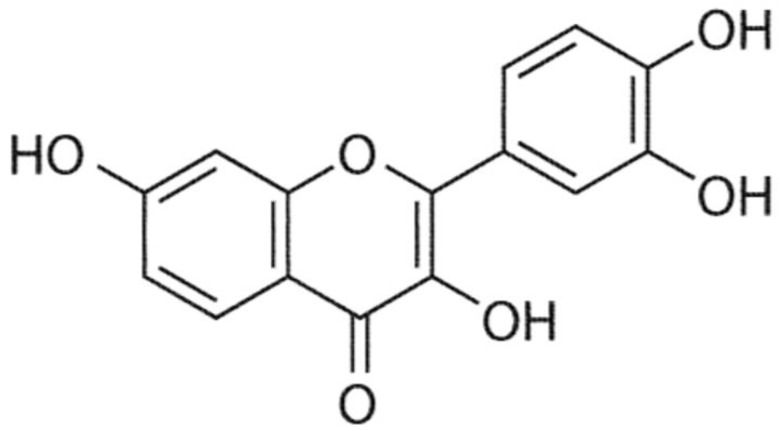
Chemical structure of fisetin.

Fisetin (3, 7, 3′, 4′-tetrahydroxyflavone) is a naturally occurring flavonoid present in various fruits, vegetables, nuts and wines [16] (Figure 1).

**Figure 2:**
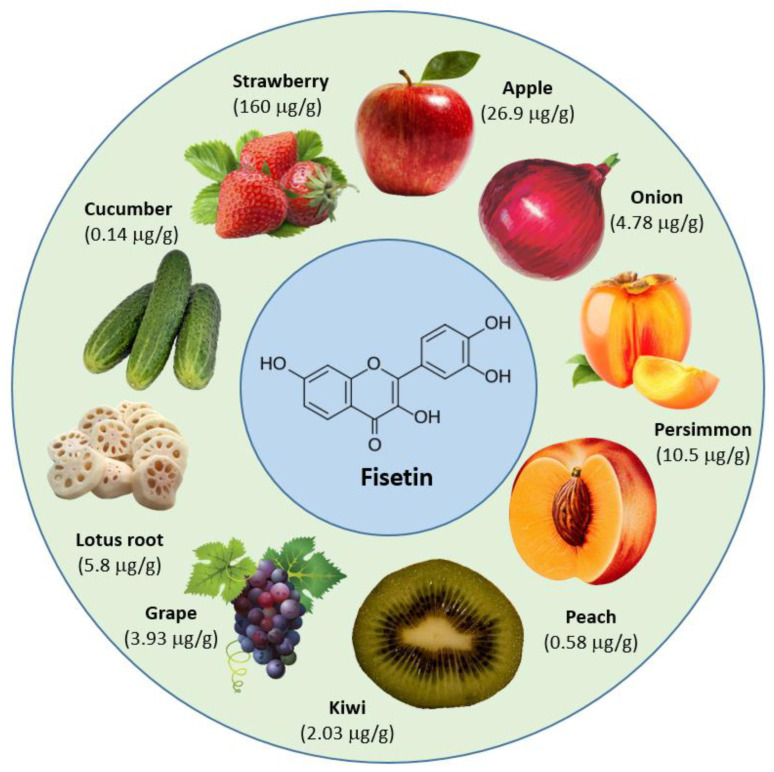
The concentration of fisetin in fruits and vegetables [17].

The concentration of fisetin is different in different types of fruits and vegetables. Strawberries (160 μg/g) showed the highest concentration of fisetin, followed by apples, with 26.9 μg/g, and persimmons, with 10.5 μg/g [17] (Figure 2).

**Figure 3:**
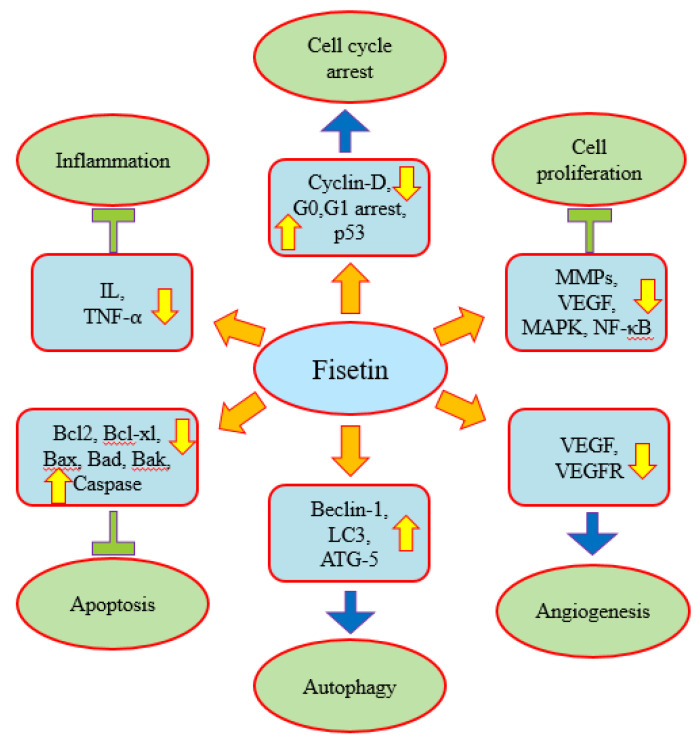
Anti-cancer potential of fisetin through the modulation of cell-signaling pathways.

Fisetin plays a role in disease management through the modulation of various biological activities. Earlier studies have demonstrated fisetin’s role in anti-oxidation, anti-inflammation and the attenuation of diabetic cardiomyopathy via the improvement of hyperglycemia/hyperlipidemia-mediated oxidative stress, inflammation as well as apoptosis [18,19,20,21]. In addition, previous studies have described fisetin’s role in different types of cancer [22,23]. The therapeutic implications of fisetin in different diseases have been documented through in vitro and in vivo studies. The anti-cancer potential of fisetin has been described through the modulation of various cell signaling pathways, including inflammation, apoptosis, angiogenesis, growth factor, transcription factor and other pathways (Figure 3).

This article summarized the anti-cancer activity of fisetin in various cancers. The synergistic effect with anticancer drugs has been described accordingly.

## 2. Anticancer Molecular Target and Mechanism-of-Action of Fisetin

The anti-cancer potential of fisetin has been confirmed through the modulation of various cell-signaling pathways. Moreover, the role of fisetin in disease management including cancer seems to be due to its anti-inflammatory antioxidant potential. It has been confirmed to target a number of cell signaling pathways including angiogenesis, apoptosis, inflammation, cell cycle, PI3K/AKT/mTOR and other pathways.

### 2.1. Inflammation

Inflammation is provoked in response to cellular damage via infection, exposure to foreign particles (irritants or pollutants) or an increase in cellular stress [24]. If the cause of inflammation continues or if certain regulatory mechanisms fail, the inflammation is converted to the chronic form [25]. This can lead to some specific mutations that contribute to the development of cancer [26,27,28]. However, the inhibition of the inflammatory process is an important step in controlling cancer development and progression. In this regard, natural products including fisetin have been proven to have anti-inflammatory potential [29,30,31,32].

In vitro- and in vivo-based results demonstrated that fisetin meaningfully improved LPS-caused inflammatory injury and oxidative stress. Moreover, some results showed that fisetin momentously inhibited the LPS-stimulated expression of TLR4 and the nuclear translocation of nuclear factor-κB (NF-κB), therefore dropping the pro-inflammatory mediators secretion. Besides silencing Nrf2, it revoked the inhibitory effects of fisetin on LPS-induced pro-inflammatory cytokines secretion, reactive oxygen species (ROS) and NADPH oxidase-4 production [33]. The effect of fisetin supplementation on the matrix metalloproteinases (MMPs) and inflammatory status levels in colorectal cancer (CRC) patients was examined. In this clinical trial, thirty-seven colorectal cancer patients undergoing chemotherapy were instructed to take either 100 mg of fisetin or placebo for seven successive weeks. Prior to chemotherapy, the supplement was given for one week and continued until the completion of the second cycle of chemotherapy. After the intervention, the plasma levels of C-reactive protein (CRP) as well as interlukin-8 (IL-8) decreased sharply in the fisetin group [34] [Table 1]. Moreover, the fisetin supplement suppressed the MMP-7 level. However, noteworthy variations were noticed only in IL-8 levels in the fisetin-treated group in comparison to the placebo group. As per the outcomes, fisetin might improve the inflammatory status in cancer patients, indicating its potential as a pioneering complementary anti-tumor agent for CRC patients [34].

### 2.2. Apoptosis

The hallmarks of cancer exist in all cancer forms regardless of the cause, such as uncontrolled growth, angiogenesis and apoptosis evasion [35,36]. Thus, natural compound-based treatment is considered very helpful in overcoming such problems. In this regard, In head and neck cancer (HSC3) cells, fisetin treatment decrease the viability as well as induced apoptosis. Through the finding from the screening of the expression profile of apoptosis-linked genes, sestrin 2 (SESN2) was functionally involved in fisetin-initiated apoptosis showing the knockdown of SESN2 by siRNA evidently restored fisetin-induced apoptosis [37].

An important study was performed to examine whether fisetin induces apoptosis in human renal carcinoma (Caki) cells. The results revealed that fisetin induced a sub-G1 population in a dose-dependent way. Additionally, the cleavage of poly(ADP-ribose) polymerase (PARP)—a substrate of caspase as well as a marker of apoptosis—was also enhanced. Fisetin caused morphological changes, followed by cell membrane blebbing and shrinkage. Thus, this finding proposed that cancer cells were more sensitive than normal cells to fisetin treatment. Fisetin induced apoptosis in SK-Hep1 cells as well [38].

An experiment was performed to examine whether fisetin treatment initiated apoptosis. The control cells of the Ca9-22 as well as the CAL-27 lines presented characteristic round nuclei, while both types of cells treated with less than 100 μM of fisetin showed condensed and fragmented nuclei and an increased ratio of nuclear condensation. Fisetin strongly decreased the mitochondrial membrane potential and the proapoptotic groups Bak and Bax. It also showed accumulation of the antiapoptotic members Bcl-XL and Bcl-2. Moreover, fisetin activated PARP and caspase-3 and produced a processed fragment dose dependently in tested cell lines [39]. A concentration of 20 and 50 µM of fisetin affected anti-apoptotic genes through the downregulation and upregulation of various pro-apoptotic genes [40]. A study reported that the apoptosis of uveal melanoma cells was prompted by fisetin efficiently. Fisetin inhibited antiapoptotic Bcl-2 family proteins as well as damaged the mitochondrial transmembrane potential. The levels of proapoptotic Bcl-2 proteins, cytochrome c, and numerous caspase activities were enhanced by fisetin [41] [Table 1].

### 2.3. Autophagy

Even though autophagy is generally supposed to be a process that alleviates numerous types of cellular stress for survival promotion, abnormal autophagy has been concerned in the pathophysiology of cancers and even causes cancer cell death [42,43,44]. It was reported that fisetin induced autophagy in pancreatic cancer cells. A western blotting-based study demonstrated that fisetin caused the upregulation of autophagy marker LC3B. Furthermore, fisetin increased the autophagic flux in cancer cells [45]. To measure whether fisetin treatment showed autophagy in human oral squamous cell carcinoma (OSCC) cells, AVO formation was measured, which is regarded as autophagic vacuoles. The results demonstrated that the development of AVO was observed after fisetin treatment in a dose-dependent fashion. Moreover, fisetin treatment enhanced the ratio of AVOs, and these outcomes indicate that fisetin treatment induces the development of autophagic vacuoles in cancer cells. Moreover, fisetin prompted autophagy in oral squamous cell carcinoma cells, which was noticed via numerous autophagy markers including Beclin-1, LC3, ATG5 as well as p62/SQSTM1 [39] [Table 1].

### 2.4. Angiogenesis

In physiological conditions, angiogenesis is a powerfully regulated process which depends on complex interactions among endothelial cells, pericytes, vascular smooth muscle cells as well as immune cells [46]. Altered angiogenesis has been noted in numerous cancers.

Natural products have confirmed their role in cancer inhibition through the control of angiogenesis. In order to determine whether fisetin affects angiogenesis in Y79 cells via the vascular endothelial growth factor (VEGF)/vascular endothelial growth factor receptor (VEGFR) pathway, VEGFR expression was noticed at the protein and mRNA levels. The results revealed that fisetin affected the expression of VEGFR, and this effect happened in a dose-dependent way. Consequently, VEGFR expression was downregulated by fisetin [47]. Fisetin inhibited capillary-like tube formation on Matrigel and migration, which were linked with a decreased expression of VEGF and endothelial nitric oxide synthase (eNOS) in human umbilical vein endothelial cells. It also decreased the expression of eNOS, inducible NOS, VEGF, MMP-2 and -9 in human cancer cells. Finally, these results indicate that fisetin inhibits various features of angiogenesis, which might support its reported antitumor effects [48].

The antiangiogenic effects of fisetin on endothelial cell migration were investigated, and it was reported that fisetin exposure at 22 and 44 μM showed a substantial dose-dependent decrease in EAhy 926 endothelial cell migration. Lewis lung carcinoma cells were mixed with Matrigel, with fisetin increasing concentrations at 44 to 350 μM, and injected subcutaneously into the right flank of mice. To measure angiogenesis, the hemoglobin content of the Matrigel plugs was examined. Fisetin treatment showed a dose-dependent decrease in Matrigel plug hemoglobin levels, which became substantial at 350 μM. These in vivo results indicate that fisetin can decrease tumor angiogenesis [49] [Table 1]. Fisetin inhibits MMP-1, 3,7,9 more proficiently than a naturally occurring MMP inhibitor, tetracycline. Fisetin inhibits the proliferation of fibrosarcoma cells, the MMP-14-mediated activation of proMMP-2 in HT-1080 cells, the invasiveness of cancer cells and human umbilical vascular endothelial cells and the in vitro tube formation of human umbilical vascular endothelial cells [50].

### 2.5. Cell Cycle Arrest

The flow cytometric analysis demonstrated that the increase in the G0/G1-phase cell population was convoyed by a concomitant decrease in the G2/M-phase and S-phase cell populations in bladder tumor cell lines. The results established that fisetin treatment caused cell cycle arrest at the G0/G1 phase and prompted apoptosis at 48 hours after treatment. The sub-G1 group meaningfully increased after the treatment of fisetin when it was compared with untreated cells; it causes 26.0% as well as 13.8% apoptotic cells at fisetin (100 μM) treatment in bladder cancer T24 as well as EJ cells, individually. Moreover, fisetin improved the expression level of p21 and p53 in a dose-dependent way. Cyclin D1, CDK2 and CDK4 were downregulated, which endorse cell cycle development via the G1 phase into the S-phase, as noticed in a dose-dependent way [51]. Another study based on leukemia reported that fisetin decreased the total viable cells via G0/G1 phase arrest and induced the sub-G1 phase. Fisetin decreased the expressions of cdc25a, whereas increased p21, p27, p-p53 and Chk1 may lead to G0/G1 phase arrest, and fisetin decreased the cell number via G0/G1 phase arrest through the induction of apoptosis through caspase-dependent and mitochondria-dependent pathways and the inhibition of cdc25c [52].

Fisetin as well as hesperetin treatment caused a concentration- and time-dependent inhibition of proliferation and induced G2/M arrest. The microarray gene profiling analysis demonstrated some significant biological pathways such as mitogen-activated protein kinases and the inhibition of DNA binding signaling pathways altered by fisetin and hesperetin treatment and provided a list of genes modulated ≥2-fold that are involved in cell proliferation, cell division as well as apoptosis [53] [Table 1].

### 2.6. PI3K/AKT/mTOR Pathway

The results based on flow cytometry as well as western blot assays indicate that fisetin administration induced apoptosis and endorsed the expression of caspase-3 by regulating Phosphoinositide-3-kinases (PI3K)/AKT/NF-κB pathways. Moreover, fisetin decreased the proliferation of laryngeal cancer cells (TU212) that were allied with the inactivation of ERK1/2. Additionally, fisetin inhibited the activation of PI3K/AKT-controlled mTOR, causing transcription suppression as well as the proliferation inhibition of laryngeal cancer cells. Finally, some findings recommend that fisetin played a vital task in the regulation of laryngeal cancer by inhibiting the proliferation of tumor cells, the induction of apoptosis as well as autophagy through ERK1/2 and AKT/NF-κB/mTOR signaling pathways [54].

Another study explained that the expressions of the JAK2, AKT and p-JAK2 proteins inoculated with fisetin showed no noticeable changes, whereas p-AKT was downregulated within the control group. Additionally, it was noticed that the total mTOR did not change; however, the p-mTOR and PI3K decreased significantly, demonstrating that the PI3K/AKT/mTOR cascade participates in the inhibition of pancreatic cancer (PANC-1) cells induced by fisetin. Consequently, fisetin may suppress the growth, invasion and migration of pancreatic cancer cells through reducing the PI3K/AKT/mTOR cascade [55]. Treatment with fisetin with a concentration of 5–20 μM caused 39–94% and 28–92% inhibition in the regulatory (p85) expression and catalytic (p110) subunits of PI3K. Fisetin also showed inhibition in the phosphorylation of Akt in lung cancer cells. Further, the enzyme-linked immunosorbent assay (ELISA)-based results showed that fisetin treatment at 5 to 20 μM caused 34 to 92% decreases, correspondingly, in the levels of p-Akt as compared to the control group in a dose-dependent fashion [56]. A recent study reported that the treatment of mammary carcinoma cells (4T1 cells) with fisetin meaningfully decreased the expression of P70, AKT and mTOR. Moreover, p-AKT and p-AKT/AKT, p-PI3K and p-PI3K/PI3K, p-P70 and p-P70/P-70 and p-mTOR were sharply decreased, upregulated Bax, and downregulated Bcl-xL following fisetin treatment as compared to the control group [57].

### 2.7. Signal Transducer and Activator of Transcription 3 (STAT3)

STAT3 plays a vital role in the regulation of important biological processes including cell proliferation, differentiation, angiogenesis, apoptosis, inflammation, invasion and metastasis [58,59]. STAT3 is the most commonly concerned protein in solid cancers [60], and in numerous types of cancer patients, an excessive activity of STAT3 is associated with poor survival outcomes [60,61,62]. Fisetin’s cytotoxic effects and inhibitory effects on cell proliferation in thyroid human cancer cells and caspases expression, along with JAK 1 and STAT3 signaling molecules, were evaluated. The findings revealed that apoptosis was induced by fisetin, which is established through reduced cell viability, improved ROS generation as well as the phases of the cell cycle. Further, JAK 1 expression and STAT3 expression were downregulated by fisetin treatment in cancer cells. Therefore, fisetin induced apoptosis in TPC-1 cancer cells via the initiation of oxidative damage and the enhancement of the caspase expression through controlling JAK 1 as well as STAT3 signaling molecules [63].

### 2.8. Wnt/β-Catenin Signaling Pathway

The overexpression of β-catenin is constitutively activated in human cancer and initiates cancer initiation, progression, metastasis, immune evasion and drug resistance [64,65]. In this regard, aiming at β-catenin signaling has been anticipated as an auspicious strategy in developing valuable anticancer agents [66,67]. A colorectal cancer-based study reported that fisetin-treated cells with various concentrations showed an inhibition of TCF4–β-catenin transcriptional activity. TOPflash luciferase activity was inhibited by 75.6% after fisetin treatment (60 μM), and the nuclear level of TCF4 decreased to some extent. This can be clarified by the substantial decrease in the nuclear β-catenin level at the same concentration of fisetin. Fisetin treatment reduced levels of Wnt-target genes including cyclin D1 and MMP-7 when treated with fisetin [68]. An important study based on human melanoma reported that a decrease in the Wnt growth factor protein was noticed in cells treated with increasing doses of fisetin. Moreover, western blot analysis indicated that increasing doses of fisetin were linked with a reduction in cytosolic β-catenin, with a concomitant reduction in nuclear β-catenin. β-catenin staining was notably reduced in the nuclei of fisetin-treated melanoma cells, indicating that a substantial amount of β-catenin was phosphorylated as well as degraded, resulting in reduced nuclear accumulation [69].

### 2.9. NF-κB Pathway

Nrf2/ARE signaling plays a crucial role in the protection against oxidative stress and is accountable for the maintenance of homeostasis as well as redox balance in cells and tissues [70].

The NF-κB pathway is significant in monitoring cell survival and proliferation, and it was involved in fisetin-initiated cell growth inhibition in bladder cancer cells. It was seen that fisetin treatment led to a substantial increase of the expression of IκBα in cancer cells. In sharp contrast, NF-κB was accumulated in the cytoplasm, and the protein expression of NF-κB in the nuclei was reduced by fisetin treatment in bladder cancer cells. It was assessed whether fisetin downregulates these NF-κB target gene products such as FLIP as well as cyclooxygenase-2 (Cox-2) by western blot analysis. It was noticed that fisetin suppressed the expression of the anti-apoptotic proteins and decreased the activity of NF-κB in a time-dependent way [ 51]. Another study based on melanoma reported that the treatment of cancer cell lines with various concentrations of fisetin played a role in the inhibition of cell invasion. BRAF-mutated melanoma cells were more sensitive to fisetin exposure, and this was linked with the phosphorylation of MEK1/2 as well as to ERK1/2 reduction. Furthermore, fisetin played a role in the inhibition of the activation of IKK and in the decrease in the activation of the NF-κB signaling pathway [71].

### 2.10. Nrf2 Pathway

Nrf2 is a cytoprotective transcription factor which demonstrated both a negative effect as well as a positive effect on cancer [72,73]. Nrf2 shows critical roles in chemotherapy resistance via stimulating the metabolism of drugs or drug efflux [74]. Fisetin translocated Nrf2 into the nucleus, and the expression of the downstream HO-1 gene was upregulated via the inhibition of the Nrf2 degradation at the post-transcriptional level [75]. A recent study reported that fisetin increased the expression of the Nrf2 protein in the nuclear fraction. The Nrf2 expression was highest at the first 4 h, and it decreased to the control level after 8 h in breast cancer (4T1) cells. Moreover, In JC cells, the Nrf2 expression was gradually increased by fisetin from 8 h to 24 h. Furthermore, by transfection siRNA against Nrf2, it was reported that fisetin-induced HO-1 protein expression was reduced through silencing Nrf2, whereas it was not affected by scrambled control siRNA in 4T1 and JC cells [76] [Table 1].

### 2.11. AP-1 pathway

AP-1 proteins play significant roles in multiple malignancies [77,78]. Fisetin upregulates ZAK expression to induce the Hippo pathway as well as to initiate the activation of JNK/ERK, the downstream of ZAK, to initiate cell apoptosis in an AP-1-dependent manner [79] (Table 1). 

## 3. Fisetin: Role in Various Types of Cancer

**Figure 4:**
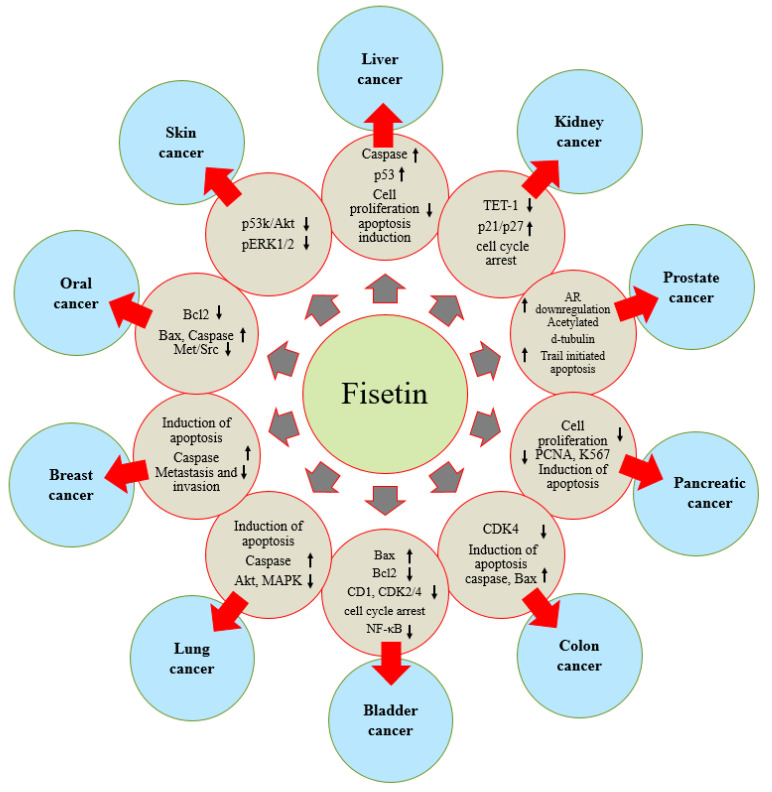
Role of fisetin in prevention of different cancers through modulation of various cell signaling molecules. Up arrow (↑) indicates upregulation, and downarrow (↓) indicates downregulation.

Fisetin has proven its role in health management, including cancer, through the modulation of various cell signaling pathways. Fisetin exerts disease prevention effects through the enhancement of the antioxidant status, the inhibition of inflammation, the induction of apoptosis and the inhibition of angiogenesis, growth factors and other genetic pathways. Its role in different types of cancers has been explained (Figure 4, Table 2).

### 3.1. Prostate Cancer

Prostate cancer is the second major cause of cancer-associated death in men in the United States [3]. Almost 80% of men who are diagnosed with prostate cancer are diagnosed with prostate-limited localized prostate cancer [80]. A large number of studies report that prostate cancer growth as well as progression are driven by the androgen receptor (AR), a ligand-dependent transcription factor as well as a nuclear receptor family member [81]. In this cancer, fisetin is a powerful, non-toxic and potent hyaluronic acid (HA) synthesis inhibitor which enhances the abundance of antiangiogenic HMM-HA and could be used for the management of this cancer [82]. A study based on an in vitro tubulin polymerization assay reported that fisetin increased microtubule polymerization to a better amount than paclitaxel under conditions that needed an enhancer for microtubules to polymerize proficiently. Additionally, it was suggested that fisetin at a similar dose showed the same effect but is faster and more effective than paclitaxel. Furthermore, whether fisetin increased acetylated α-tubulin, which is associated with increased microtubule stability, was examined. It was reported that treatment of DU145 cancer cells and PC-3 cells with fisetin at a dose of 20–80 μM encourages the high expression of acetylated α-tubulin, as compared with β-actin and α-tubulin as the control. These outcomes indicate that the treatment of fisetin stabilizes microtubules and affects the modification of post-translational tubulin [83].

Another experiment was planned to observe the effect of fisetin on the tumor necrosis factor (TNF)-related apoptosis-inducing ligand-induced apoptosis potential in prostate cancer cells. Finally, a finding revealed that fisetin sensitizes the TNF-related apoptosis-inducing ligand-resistant androgen-dependent LNCaP as well as the androgen-independent PC3 and DU145 prostate cancer cells to TNF-related apoptosis-inducing ligand-induced death. Moreover, fisetin increased TRAIL-initiated apoptosis and cytotoxicity in cancer LNCaP cells via appealing the intrinsic (mitochondrial) and extrinsic (receptor-mediated) apoptotic pathways [84]. The effect of fisetin on the hormone-independent cell lines PC3 and DU145 was investigated. PC3 cancer cells revealed less sensitivity to fisetin as compared to DU145. Moreover, the activity of the mTOR kinase in PC3 cells treated with fisetin was assayed. The activity of mTOR was inhibited by 6, 11 and 25% when the cells were treated with various concentrations of fisetin. Overall, based on the findings, this study suggests that fisetin acts as a dual inhibitor of mTORC1/2 signaling, leading to the induction of autophagic cell death as well as the inhibition of Cap-dependent translation in prostate cancer cells [85].

### 3.2. Kidney Cancer

Kidney cancer ranks as the seventh as well as tenth most common cancer in men and women, correspondingly, in the United States [86]. An important study was performed to examine fisetin’s role in renal cancer cells. The results confirmed that treatment with fisetin induced a sub-G1 population in a dose-dependent fashion. Additionally, fisetin caused cell shrinkage as well as membrane blebbing. Consequently, this finding indicates that cancer cells were highly sensitive, compared to normal cells, to fisetin treatment. Furthermore, DNA fragmentation and chromosomal damage were noticed in fisetin-treated cells [38]. Fisetin-caused apoptosis, dependent upon the enhancement of caspase, was investigated, and fisetin evidently enhanced the caspase stimulation. Furthermore, a pan-caspase inhibitor such as z-VAD-fmk totally stopped the fisetin-induced sub-G1 population as well as PARP cleavage. This result indicated that the fisetin-caused caspase facilitated apoptosis [86].

Fisetin inhibited cell viability via cell cycle arrest in the G2/M phase, further upregulating p21/p27 and downregulating cyclin D1. Fisetin inhibited the migration as well as the invasion of human renal cell carcinoma (RCC) cells via the downregulation of CTSS and MMP-9 (ADAM9) and disintegrin. Additionally, fisetin upregulated ERK phosphorylation in Caki-1and 786-O cells. Fisetin inhibits the proliferation and metastasis of RCC by downregulating ADAM9 and CTSS through the signaling pathway of MEK/ERK [87]. Fisetin can sharply decrease the expression levels of TET1 in kidney renal stem cells (HuRCSCs) and 5hmC levels. Together, the ChIP-PCR findings revealed that fisetin can efficiently block 5hmC modification levels at the CpG islands in cyclin Y as well as CDK16 and reduce their transcription and activity. Therefore, it was concluded that fisetin inhibits the epigenetic mechanism in renal cancer stem cells [88].

### 3.3. Liver Cancer

Fisetin reduced autophagic flux formation in a dose-dependent way. In the gene expression analysis, the mRNA levels of mTOR, Atg16L, Atg5 and LC3A were increased, while the mRNA levels of Beclin1 and Atg7 were decreased compared to the control. Moreover, fisetin treatment inhibited the expression of mTOR, Atg7, Atg16L and pACC and elevated the expression of AMPKα, Atg5, ACC, AMPKβ1/2 and Akt. Overall, the results demonstrated that fisetin inhibited autophagy via the activation of PI3K/Akt/mTOR and modulated the AMPK signaling pathways [89].

In order to discover whether fisetin might be a death receptor 2 (DR2) agonist showing a vital task in regulating the proliferation and viability of liver cancer cells, fisetin was given to liver cancer (HCC-LM3) cells to examine the effects. Fisetin displayed a suppressing role in HCC-LM3 proliferation. Likewise, the death receptor-2 agonist of bromocriptine suppressed HCC-LM3 cell proliferation. Overall, the results indicated that fisetin plays similar role as bromocriptine, i.e., decreasing liver cancer cell lines proliferation, which might be a vital therapeutic approach for liver cancer treatment, associated with the DR2 signal [90].

### 3.4. Colon Cancer

DNA synthesis and cell growth were blocked by fisetin treatment; a disturbed cell cycle progression from the G1 phase to the S phase was noticed at eight hours, and G2/M phase arrest was noticed at twenty-four hours. Moreover, the activities of cyclin-dependent kinases (CDK) 2 as well as CDK4 were decreased by fisetin and also inhibited CDK4 activity in a cell-free system, demonstrating that it might directly inhibit the activity of CDK4. Cell division cycle 2 (CDC 2) as well as CDC25C protein levels and the CDC2 activity were decreased in fisetin-treated cells [91].

A study was performed to examine whether fisetin was capable of sensitizing both Irinotecan as well as Oxaliplatin resistance colon cancer cells and discovered the probable signaling pathways involved using in vitro as well as in vivo models. The outcomes indicated that fisetin treatment efficiently inhibited cell viability as well as the apoptosis of CPT11-LoVo cells compared to Oxaliplatin and parental LoVo cancer cells. Moreover, apoptosis was encouraged by fisetin treatment, endorsing caspase-8 and cytochrome-c expressions probably via the inhibition of the aberrant activation of IGF1R as well as AKT proteins. Fisetin inhibited tumor growth in an athymic nude mouse xenograft model. In total, the outcomes provide a basis for fisetin as a promising agent to treat parental as well as chemoresistance colon cancer [92].

The treatment of cancer cells with fisetin showed an increase in apoptosis, and the treatment of cells with 120 μM of fisetin-treated cells showed late-stage apoptosis. Likewise, caspase-3 was cleaved and therefore activated in cells treated with fisetin. The treatment of cells with fisetin increased the level of the proapoptotic Bak protein and decreased the levels of the prosurvival Bcl-2 and Bcl-XL proteins, and the Bax level was not changed after the treatment of fisetin [92]. An important study tried to describe the mechanisms by which fisetin causes apoptosis in HCT-116 cells. Fisetin caused an increase in the levels of proapoptotic Bim and Bak and induced a reduction in the protein levels of antiapoptotic Bcl-2 and Bcl-xL, and bax was unchanged. Moreover, release of cytochrome c and Smac/Diablo was induced by fisetin, and it also increased the permeability of the mitochondrial membrane. Fisetin caused an increase in the protein levels of cleaved caspase-8, DR5, Fas ligand, and TNF-related apoptosis-inducing ligand, and the caspase-8 inhibitor Z-IETD-FMK decreased fisetin-induced apoptosis as well as the activation of caspase-3. This result confirms that fisetin induces apoptosis in HCT-116 cells through the activation of the death receptor as well as the mitochondrial-dependent pathway, followed by the activation of the caspase cascade [93].

Fisetin-induced apoptosis in HCT116 cells caused an increase in HCT116 securin-null cells or in wild-type cells, and securin was knocked down via siRNA but reduced when wild-type or non-degradable securin was reconstituted. Furthermore, fisetin did not induce apoptosis in HT-29 p53-mutant cells and the HCT116 p53-null group. This result, for the first time, suggests that securin reduction sensitizes human colon cancer cells to fisetin-induced apoptosis [94].

### 3.5. Gastric Cancer

A gastric cancer cells-based study reported that fisetin (less than 25–100 µM) showed a substantial decrease in G1 phase cyclins and CDK levels, and the levels of p53 were enhanced. Additionally, the death of non-neoplastic FHs74int as well as the growth suppression were nominally changed by the treatment of fisetin. Fisetin powerfully improved apoptotic cells and caused the depolarization of the mitochondrial membrane. Fisetin played a role in the induction of apoptosis, independently of p53, and increased mitochondrial ROS generation. Overall, these findings confirmed that fisetin holds anticancer potential via ROS creation, most probably via the MRC complex I causing gastric carcinoma cells apoptosis [95]. Gastric cancer cells such as GES-1 and SGC7901 cells were exposed with various concentrations of fisetin (1 to 20 µM) and suggestively decreased the proliferation rate of SGC7901 cells from 98% to 11%, correspondingly, as compared to the control (100%) after 48 h. At 1 to 20 µM concentrations of fisetin, the proliferation rate of GES-1 cells was found to be 100 to 98%, respectively. Moreover, the treatment of SGC7901 cells with various concentrations of fisetin induced cell death in a dose-dependent fashion [96].

### 3.6. Pancreatic Cancer

A study was performed to explore the combined effect of fisetin and gemcitabine on human pancreatic cancer cells. Gemcitabine as well as fisetin combination treatment suppressed/inhibited the proliferation of pancreatic cancer cells as well as encouraged apoptosis, as indicated by caspase 3/7 activation. Decreased ERK-initiated MYC instability at the protein level as well as the downregulation of ERK at the protein and mRNA levels were seen after fisetin treatment. Finally, the study concluded that fisetin played a role in the sensitization of human pancreatic cancer cells to gemcitabine and caused cytotoxicity by ERK-MYC signaling inhibition. These findings indicate that the combination of gemcitabine and fisetin could be established as an innovative and powerful therapeutic agent [97].

To examine the effect of fisetin on pancreatic ductal adenocarcinoma cells, the pancreatic cancer cells PANC-1 and BxPC-3 were treated with increasing concentrations of fisetin. Remarkably, it was found that low concentrations of fisetin (25 to 50 µM) did not inhibit the viability of PANC-1 cells proficiently, whereas, at same time, BxPC-3 cells were sensitive with low concentrations of fisetin. PANC-1 cells were cultured with different concentrations of fisetin, and the findings exhibited that fisetin reduced PANC-1 cell viability. These findings indicated that the proliferation of PANC-1 cells was blocked by fisetin. In addition, the in vivo effect of fisetin was investigated using a xenograft nude mouse model of luciferase-expressing human pancreatic PANC-1 tumor cells. Remarkably, tumor sizes were meaningfully decreased in fisetin-treated mice. Fisetin decreased proliferation-related proteins such as PCNA, Ki67 and phosphorylated histone H3 (p-H3) and decreased the expression of cell growth [45].

A recent study reported that fisetin played a role in the enhancement of DSBs through the ZC3Hl3-mediated m^6^A modification of PHF10, which may improve the novelty in therapeutic approaches for pancreatic ductal adenocarcinoma [98]. Fisetin treatment showed the inhibition of pancreatic cancer cell growth and cell proliferation with associated apoptosis induction. Moreover, fisetin modulates the expression of more than twenty genes at the transcription level, and a parallel increase can be seen in the expression levels of an NF-kB inhibitor, I𝜅B⍺. The transient downregulation of DR3 by RNA interference meaningfully increased fisetin-induced changes in cell proliferation, cell invasion and apoptosis. These data suggest that fisetin could offer a biological rationale for the treatment of pancreatic cancer [99]. (Figure 4)

### 3.7. Bile Duct Cancer

An interesting study was conducted to examine the anti-cancer potential of fisetin treatment in association with gemcitabine. The cytotoxic effect of gemcitabine as well as fisetin treatment on a human cholangiocarcinoma cell line (SNU-308) was measured. Fisetin inhibited the survival of cholangiocarcinoma cells by powerfully phosphorylating ERK. It also brought cellular apoptosis in combination with gemcitabine. Fisetin treatment played a role in the reduction of Phospho-p65 and myelocytomatosis expression. These findings recommend fisetin in combination with gemcitabine as an option for better-quality anticancer regimens [100].

### 3.8. Bladder Cancer

Fisetin caused the induction of apoptosis in bladder cancer, and it is initiated through the regulation of two related pathways, the downregulation of the NF-κB pathway and the upregulation of p53 activity, initiating changes in the ratio of pro- and antiapoptotic proteins. In the meantime, the treatment of fisetin suggestively decreases the MNU-induced bladder tumor incidence via decreasing the activation of NF-κB and modulating the NF-κB expression target genes that control cell apoptosis and cell proliferation. This finding suggests that the inhibition of the NF-κB pathway and the activation of p53 might show imperative roles in the fisetin-initiated apoptosis in bladder cancer. Intravesical fisetin excellently inhibited bladder cancer carcinogenesis in MNU-caused rats. These results recognize the in vivo chemo-preventive effectiveness of fisetin, and the results of the study indicate that fisetin might be used as an innovative, efficient and harmless intravesical agent for bladder cancer [101].

The proliferation of EJ and T24 cells was inhibited by fisetin via stopping cell cycle progression in the G0/G1 phase and caused the induction of apoptosis. The western blot assay exhibited that fisetin increases the expression of p21 and p53 proteins and decreases the levels of cyclin A, cyclin D1 as well as CDK2,4 thus contributing to the arrest of the cell cycle. Moreover, fisetin increased the expression of Bak as well as Bax but decreased the levels of Bcl-xL and Bcl-2 and triggered the mitochondrial apoptotic pathway. This finding indicates that the inhibition of NF-κB and the stimulation of p53 might play imperative roles in the fisetin-initiated apoptosis in bladder cancer cells [51].

### 3.9. Lung *Cancer*

Lung cancer is the second most common cancer in both men as well as women and is the foremost cause of cancer mortality in the United States [102]

Treatment with fisetin played a role in the inhibition of the growth and migration of non-small cell lung cancer (NSCLC), and this was confirmed by an in vitro-based study. Proliferation, adhesion, migration and invasion were suppressed by fisetin. Flow cytometry-based results indicated that fisetin treatment induces apoptosis in the cancer cell line via reducing the expression of cyclin-D, c-myc, B cell lymphoma-2, COX-2, MMP-2,-9 and cluster of differentiation (CD) 44 and enhancing the expression of CDK inhibitor 1A/B, E-cadherin and CDKN2D, and caspase-3/9 activity increased through directing the extracellular signal-regulated kinase signaling pathway [103].

The combination of fisetin and paclitaxel meaningfully reduced cancer cell migration as well as invasion, at least somewhat, via a noticeable rearrangement of the cytoskeleton of vimentin and actin and metastasis-associated gene modulation. Most of this activity of the mixture treatment was considerably higher than that of separate agents. Paclitaxel treatment only showed more toxicity to normal cells than the combination of flavonoids with paclitaxel, suggesting that fisetin might bring some safety against paclitaxel-facilitated cytotoxicity. The combination of fisetin and paclitaxel probably shows a synergistic anti-cancer effectiveness and is an important power for the treatment of human NSCLC [104].

A study was performed to explore the anti-cancer activity that might perform synergistically with an anti-cancer drug (paclitaxel) to produce growth prevention and/or pro-death effects on lung cancer cells (A549). The results confirm the presence of synergism between fisetin as well as paclitaxel treatment in the lung cancer based on an in vitro model. The switch from the cytoprotective autophagy to the autophagic cell death was also concerned in the method of the synergistic action of the cancer drug (paclitaxel) as well as fisetin in the lung cancer cells (A549). Moreover, the synergism between paclitaxel and fisetin was cell-line-precise, fisetin synergizes with arsenic trioxide, whereas such effects were not seen with methotrexate and mitoxantrone in the lung cancer cells (A549) [105].

Erlotinib-resistant lung adenocarcinoma cells (HCC827-ER), cultured from the cell line HCC827, were studied to determine the role of erlotinib as well as fisetin on the cell viability as well as apoptosis. The study showed that fisetin efficiently enhanced the sensitivity of erlotinib-resistant lung cancer cells to erlotinib, probably via preventing the abnormal activation of AKT as well as MAPK signaling pathways brought about from the suppression of AXL. Overall, fisetin probably reversed the attained erlotinib resistance of lung cancer [106]. Fisetin induced mitochondrial ROS and distinctive signs of ER stress: ER staining, the expression of ER stress-related proteins, mitochondrial Ca^2+^ overload glucose-regulated protein (GRP)-78, the cleavage of triggering transcription factor 6, the phosphorylation of the eukaryotic initiation factor-2α subunit, the splicing of X-box transcription factor-1 and the initiation of C/EBP homologous protein and cleaved caspase-12.

Furthermore, fisetin induced the phosphorylation of p38 MAPK, ERK and JNK [107]. Fisetin inhibits cell growth with the concomitant inhibition of mTOR as well as PI3K/AKT signaling in NSCLC cells. Fisetin interrelates with the mTOR complex through two sites. Treatment with fisetin showed the reduction in the formation of lung cancer A549 cell colonies in a dose-dependent fashion. The treatment of cells with fisetin showed the inhibition of the phosphorylation of Akt, 4E-BP1, eIF-4E, mTOR and p70S6K1 and a decrease in the protein expression of PI3K. Cells treated with fisetin demonstrated the inhibition of the constituents of the mTOR signaling complex. It was noticed that the treatment of cells with the mTOR inhibitor rapamycin as well as mTOR-siRNA caused a decrease in the phosphorylation of mTOR as well as its target proteins that were additionally decreased in fisetin treatment [56].

### 3.10. Skin Cancer

Melanomas are caused by genetic predisposition ad other phenotypic factors includes fair skin as well as many moles [108]. Various natural compounds have proven role in cancer including melanoma.

Fisetin-loaded binary ethosomes were made, and its role in skin cancer was evaluated. The prepared formulations were examined for entrapment efficiency, vesicle size as well as the flux of fisetin. An in vivo-based study was conducted to examine the tumor incidence, lipid peroxidation values, glutathione content, pro-inflammatory cytokines and catalase activity in mice. The optimized binary ethosomes formulation showed sealed unilamellar-shaped vesicles, with a vesicles size, entrapment efficiency and flux of 99.89 ± 3.24 nm, 89.23 ± 2.13% and 1.01 ± 0.03 µg/cm^2^/h, respectively. The in vivo study showed that the mice previously treated with this formulation gel showed a noticeable decrease in the levels of pro-inflammatory markers including IL-1α and TNF-α in comparison to the mice exposed to UV only. These formulation gel-treated mice showed a lower percentage of tumor incidences (49%) in comparison to mice treated with ultra violet only (tumor incidence: 96%). Finally, the fisetin-loaded binary ethosomes formulation works as a powerful dermal delivery system for controlling skin cancer [109].

Various concentrations of fisetin were used to treat multiple human malignant melanoma cell lines, and the results demonstrated that fisetin treatment caused the inhibition of cell invasion. BRAF-mutated melanoma cells were extremely vulnerable to fisetin treatment, and this was related to a reduction in the phosphorylation of ERK1/2 as well as MEK1/2. Furthermore, fisetin inhibited the IKK, causing a decrease in the activation of the NF-κB signaling pathway. Additionally, the treatment of fisetin endorsed mesenchymal-to-epithelial transition in cancer cells, which is linked to a decrease in mesenchymal markers as well as an increase in epithelial markers. These outcomes indicate that fisetin prevents melanoma cell invasion through the promotion of mesenchymal-to-epithelial transition and by targeting NF-κB and mitogen-activated protein kinase (MAPK) signaling pathways [71]. A recent pioneer study reported that fisetin decreased the cell viability of skin cancer cell lines (A375 and A431) in a dose- and time-dependent way. Moreover, fisetin meaningfully decreased the colony formation as well as the migratory capability of the cancer cells. The results based on the flow cytometry analysis indicated that fisetin significantly limited the progression of skin cancer cells in the G0/G1 phase of the cell cycle and induced cells to undergo apoptosis via decreasing the mitochondrial membrane potential, increasing ROS and elevating the count of early and late apoptotic cells [110].

Fisetin treatment to ultraviolet B (UVB)-exposed mice decreased the hyperplasia condition and decreased inflammatory cells. Disetin treatment decreased inflammatory mediators including prostaglandin E2 as well as its receptors (EP1-EP4), cyclooxygenase-2 and myeloperoxidase action. Additionally, fisetin decreased the levels of inflammatory cytokines, IL-1β, IL-6 and TNF-α in UVB-exposed skin. The treatment of fisetin decreased cell proliferation markers and DNA damage, as demonstrated by the increased expression of p21 and p53 proteins. Additional findings showed that fisetin inhibited the UVB-induced expression of the phosphorylation of PI3K and AKT and the enhancement of the NF-κB signaling pathway [111].

### 3.11. Oral Cancer

Fisetin brought cell death via the cell morphological changes, arrested the G2/M phase, promoted ROS and the production of Ca^2+^, induced apoptosis, increased the caspase and reduced the level of the mitochondria membrane potential in human oral cancer cells. Fisetin induced cell apoptosis in SCC-4 cells, decreased the anti-apoptotic proteins including Bcl-2 and increased the proapoptotic proteins such as Bid and Bax. Moreover, the finding also indicated that fisetin in cancer cells enhanced the cytochrome c, AIF as well as Endo G release from the mitochondria. Based on those explanations, it was suggested that fisetin initiated cell apoptosis via mitochondria, ER stress as well as caspase-dependent pathways [112].

Fisetin encouraged apoptotic cell death via increased ROS and Ca^2+^, while it increased caspase-8, -9 and -3 activities and reduced the mitochondrial membrane potential in HSC3 cells. Additionally, fisetin induced chromatin condensation and induced DNA damage in HSC3 cells. Fisetin reduced the anti-apoptotic proteins, increased the expression of pro-apoptotic proteins and enhanced the cleaved forms of caspase, cytochrome c, apoptosis-inducing factor as well as endonuclease G [113]. An important study reported that fisetin expressively inhibits tumor cell proliferation and initiates apoptosis in oral SCC cell lines. Additional findings establish that fisetin inhibits Met/Src signaling pathways and reduces the basal expression of Src as well as the Met protein in cancer cell lines (UM-SCC-23). Overall, these findings deliver innovative visions into the action of fisetin and propose probable therapeutic approaches for oral carcinoma via blocking the Met/Src signaling pathways [114].

Fisetin inhibited the survival rate of CNE-LMP1 cells, and NF-κB activation was caused by latent membrane protein-1. Fisetin also repressed IκBα phosphorylation and the nuclear translocation of NF-κB (p65) and inhibited Cyclin D1, all key objects of the NF-κB signal transduction pathway. It was suggested that the interference effects of fisetin with signal transduction activated via latent membrane protein-1 encoded by the Epstein-Barr virus may show an imperative role in its anticancer potential [115]. The dietary flavonoid fisetin indicated that apoptosis was induced by the treatment of fisetin, endorsing the expression of caspase-3 through the control of PI3K/AKT/NF-κB. Furthermore, fisetin suppressed cancer cell TU212 cells proliferation, which was associated with the deactivation of ERK1/2. Additionally, fisetin treatment caused the inhibition of the enhancement of PI3K/AKT and controlled mTOR, leading to proliferation inhibition and TU212 cells transcription suppression. In addition, in vivo-based finding also indicated that the tumor volume as well as the weight of nude mice were reduced due to fisetin treatment, with a decrease in KI-67 as well as an enhancement in LC3II in tumor tissue samples [54].

### 3.12. Leukemia

Leukemia incidence rate is high (about 27,600 per 100,000 people) because of its varied pathogenic factors [116]. 

Fisetin played a role in killing THP-1 cells in vivo and resulted in a tumor decrease in the xenograft mouse model. Death initiation in vitro was facilitated by an increase in nitrite, causing the enhancement of both the extrinsic as well as the intrinsic apoptotic pathways and breaks of the double-strand DNA. Fisetin inhibited the downstream components of the mTORC1 pathway via inducing the hypo-phosphorylation of S6 Ri P kinase, eIF4B and eEF2K. The downregulation of the levels of p70 S6 kinase and NO inhibition returned the phosphorylation of downstream effectors of mTORC1 and rescued the cells from death. Fisetin encouraged Ca^2+^ entry via L-type Ca^2+^ channels, and the abrogation of Ca^2+^ influx reduced caspase activation as well as cell death [117].

### 3.13. Breast Cancer

Apoptosis in caspase-3-deficient MCF-7 cells was activated by fisetin and categorized by numerous apoptotic structures. This was in conjunction with the depolarization of the mitochondria, the breakdown of the plasma membrane, the stimulation of caspase-7(-9) and the cleavage of PARP, although neither phosphatidylserine externalization nor the fragmentation of DNA were seen. While p53 was activated by fisetin treatment, the fisetin-induced apoptosis was not rescued by the pifithrin-α, a p53 inhibitor. Moreover, the fisetin-induced apoptosis was revoked by z-VAD-fmk, a pan-caspase inhibitor. Additionally, the inhibition of autophagy using fisetin was revealed as an extra route to speedy anticancer activity in MCF-7 cells [118]. An interesting study was conducted to evaluate the anti-invasive potential of fisetin based on breast cancer cells.

Fisetin decreased 12-O-tetradecanoylphorbol-13-acetate (TPA), initiated cell invasion in human breast cancer cells (MCF-7) and was seen to inhibit the enhancement of the p38 MAPK as well as the PKCα/ERK1/2/ROS signaling pathways. This potential was also linked with a decrease in NF-κB activation, signifying the anti-invasive potential of fisetin on human breast cancer cells (MCF-7). It might arise from a decrease in the TPA activation of the p38 MAPK and PKCα/ERK1/2/ROS signals, and it inhibited the TPA activation of NF-κB, finally leading to the MMP-9 expression downregulation. The results indicate the role of fisetin in MCF-7 cell invasion and elucidate the causal molecular mechanisms of this role, demonstrating that fisetin is a potential chemo-preventive agent for metastasis breast cancer [119].

Fisetin inhibited the growth of MDA-MB-231 and MDA-MB-468 triple-negative breast cancer cells, in addition to their capability to form colonies, without significantly affecting the non-malignant cell growth. Additionally, EGFR-2-over-expressed SK-BR-3 breast cancer cells, and the growth of estrogen receptor-bearing MCF-7 breast cancer cells was inhibited by the treatment of fisetin. Fisetin inhibited triple-negative breast cancer cell division and induced apoptosis, which was related with the stimulation of the caspase (9 and 8), the cleavage of poly (ADP-ribose) polymerase-1 and the permeabilization of the mitochondrial membrane.

The treatment of fisetin played a role in the induction of caspase-dependent apoptosis by decreasing the killing of triple-negative breast cancer cells. The reduced phosphorylation of histone H3 at serine 10 in the treatment of fisetin triple-negative breast cancer cells at the G2/M phase of the cell cycle indicated that fisetin-initiated apoptosis was the effect of the prevention of Aurora B kinase [120]. Another study discovered the anti-cancer potential of fisetin in mammary carcinoma cells, and its fundamental mechanisms were revealed. The finding suggests that fisetin suppressed the metastasis as well as the invasiveness of 4T1 cells, repressed the proliferation of breast cancer cells and brought about the apoptosis of 4T1 cells, and these findings were reported based on an in vitro study. The in vivo-based findings established that fisetin enhanced tumor cell apoptosis and suppressed the growth of 4T1 cell-derived orthotopic breast tumors. The current outcome indicates that fisetin showed an anti-mammary carcinoma effect [57]. A recent pioneer study reported that fisetin induced the apoptosis of breast cancer cells by numerous mechanisms, including the induction of proteasomal degradation, decreasing its half-life, the inactivation of the receptor, decreasing enolase phosphorylation, as well as the alteration of PI3 kinase/AKT signaling [121].

### 3.14. Ovarian Cancer

An interesting study was conducted to examine the role of fisetin and its nanoparticles (NPs) on apoptosis induction and the anti-proliferation of the human ovarian cancer cell line SKOV3. The MTT test indicated that fisetin as well as fisetin NPs had an inhibitory effect on ovarian cancer cells. After the treatment of fisetin and its NPs, the heart, kidney, spleen, liver and lung were found to be safe, without damage. Fisetin and fisetin NPs have the role in anti-ovarian cancer cells, and no organ injury was found [122]. After the fisetin treatment, the chromatin was taken together, and the apoptotic bodies were noticed in the ovarian cancer cell line (SKOV3). The MTT assay-based findings indicated that fisetin played a role in the inhibition of the proliferation of ovarian cancer cells in a dose-dependent manner. Moreover, after fisetin treatment, apoptosis was induced in SKOV3 cells.

In an athymic nude rat model, the tumor mass as well as the tumor volume were reduced under the effect of fisetin. The western blot-based results indicated that treatment with high concentrations of fisetin caused a substantial increase in Bax, whereas it caused a decrease in Bcl-2. The outcomes delivered insight into the induction of apoptosis and the anti-tumor and anti-proliferative effectiveness of fisetin against ovarian cancer based on in vitro as well as in vivo findings [123]. To examine the cytotoxic effects of fisetin/fisetin micelles on human ovarian cancer cells, cancer cells were treated with increasing concentrations of fisetin/fisetin micelles, and the anti-proliferation effect was tested after different time durations. The numbers of cells were reduced after the fisetin/fisetin micelles treatment in a time- and dose-dependent fashion in cancer cells, whereas this was not noticed in normal cell lines [124].

### 3.15. Cervix Cancer

The combination of fisetin and sorafenib synergistically encouraged apoptosis in HeLa cancer cells, which is convoyed by a noticeable rise in the loss of mitochondrial membrane potential. Apoptosis induction was accomplished by the activation of caspase-3, 8, which enhances the Bcl-2/Bax ratio and caused the cleavage of the PARP level, whereas it altered the mitochondrial membrane potential in cervix cancer HeLa cells. Moreover, the combined sorafenib and fisetin treatment showed significantly higher activity than the sorafenib treatment alone, and this result was confirmed by in vivo-based findings. In vivo- and in vitro-based results demonstrated that the combination of fisetin and sorafenib showed better synergistic effects than the individual compound used against cervical cancer [125].

Fisetin induced the apoptosis of cervix cancer HeLa cells, and this polyphenol played a role in the activation of caspases-3, -8 and the cleavage of poly (ADP-ribose) polymerase, resulting in the initiation of apoptosis. Likewise, cervix cancer HeLa cells treated with fisetin encouraged a continuous activation of the phosphorylation of ERK1/2, and the inhibition of ERK1/2 or the transfection with the mutant ERK1/2 expression vector suggestively eliminated the fisetin-initiated apoptosis via the activation of the caspase-8, -3 pathway. The in vivo xenograft mice-based findings revealed that fisetin meaningfully decreased the tumor growth in mice with HeLa tumor xenografts [126].

Fisetin (40 µM) was not knowingly toxic to CaSki and SiHa cells for 24 to 48 h. In the cell migration assay, cervix cancer SiHa cells treated with fisetin concentrations (20 and 40 µM) exhibited a decrease in motility of 46.0% as well as 81.3%, and a similar finding was also seen in CaSki cells, with 62.1% and 90.2% of inhibition, respectively.

Fisetin was revealed to decrease cell invasion in a concentration-dependent fashion. After fisetin treatment at 40 µM, invasion was reduced by 87.2% and 92.4%, whereas after fisetin treatment at 20 µM, invasion was decreased by 52.4% and 59.4% in SiHa and CaSki cells, respectively. These results indicated that fisetin inhibited the migration and invasion of cervical cancer cells under non-toxic concentrations. Fisetin blocked the TPA (tetradecanoylphorbol-13-acetate)-caused inhibition, the TPA-enhanced migratory and invasive abilities and the activation of p38 MAPK and uPA. Additionally, the promoter activity of the uPA gene was intensely suppressed by fisetin, which disrupted NF-κB as well as its binding quantity on the promoter of the uPA gene [127].

### 3.16. Endometrial Cancer

The study was performed on human endometrial cancer cells to examine the anti-proliferative effect of fisetin. Fisetin (20–100 µM) efficiently decreased the viability of KLE as well as Hec1 A cells and possibly changed the population of cells at the G2/M stage. Fisetin suppressed the expression of cyclin B1, triggered the inactivation of Cdc2 and Cdc25C by increasing their phosphorylation levels and additionally activated Chk1, 2 and ATM. Enhanced levels of p27 and p21 were also seen. This study proposes that fisetin caused the arrest of the G2/M cell cycle via deactivating Cdc25c as well Cdc2 via the activation of Chk1, 2 and ATM [128].

### 3.17. Osteosarcoma

A recent study on osteosarcoma reported that fisetin decreased the viability of cells at various concentrations. Fisetin suppressed migration, cell mobility and invasion and inhibited MMP-2 activity in U-2 OS cells. Moreover, western blotting indicated that fisetin decreases the levels of SOS-1, pEGFR, GRB2, PKC, Ras, p-p-38, p-ERK1/2, p-JNK, VEGF, FAK, PI3K, RhoA, p-AKT, uPA, NF-ĸB, MMP-7,-9 and -13, whereas it increases GSK3β as well as E-cadherin in U-2 OS cells after 48 h of treatment. Overall, fisetin could be used as a target for the treatment of the metastasis of osteosarcoma cells [129].

Fisetin treatment was related with the decrease in colony formation in cancer cells such as U2OS, whereas it not observed in MG63 cells and MG63 cells, and Saos-2 cells showed decreased cell proliferation after fisetin treatment. Furthermore, fisetin treatment (50 µM) for 48 h caused the arrest of the cell cycle G2-phase. Irrespective of the combination, etoposide and fisetin decreased the percentage of cells in the G1 phase and increased the percentage of cells in the G2 phase. Mixtures with additional positive combined effects induced the increased percentage of cells in the S-phase. When compared to etoposide treatment only, these combinations showed reduced levels of the cyclins B1 and E1 [130]. Another study reported that fisetin efficiently decreased the viability of osteosarcoma cells, induced apoptosis by suggestively inducing the expression of caspases 3,8,9, Bax and Bad (pro-apoptotic proteins) and downregulated Bcl-2 and Bcl-xL, whereas fisetin treatment caused the inhibition of the ERK1/2 and PI3K/Akt pathways. Fisetin induced ROS generation as well as a reduction in the mitochondrial membrane potential, which would have contributed to an increase in apoptotic cell counts [131].

### 3.18. Brain Cancer

Fisetin, a naturally occurring flavonoid, displayed the effective inhibition of cell migration as well as the inhibition of the invasion of GBM8401 cells. The expression of the ADAM9 protein as well as mRNA was inhibited by fisetin treatment. Fisetin phosphorylated ERK1/2 in a continued way, which helped in the inhibition of the ADAM9 protein as well as the expression of mRNA, which was noticed by RT-PCR as well as the Western blot. Furthermore, ERK1/2 inhibition through U0126 or transfection with the siERK plasmid eliminated the fisetin-inhibited migration as well as the invasion via ERK1/2 pathway activation [132]. The antiproliferative effects of fisetin in T98G and BEAS-2B cells were examined by an MTT assay. Therefore, fisetin was determined to have more of an apoptotic potential in T98G than BEAS-2B cells. Furthermore, fisetin was found to have cytotoxicity at lower doses in T98G cells compared to the positive control, carmustine. Caspase -8,-9 and the expressions of BAX were enhanced by the used fisetin doses of 25 and 50 μM, whereas those of survivin and BCL-2 were reduced in T98G cells [133].

### 3.19. Retinoblastoma

Retinoblastoma, the most common primary malignant intraocular tumor in children, affects 1 in 16,000 to 18,000 live births [134].

An important study based on retinoblastoma reported that fisetin significantly inhibited the proliferation of Y79 cancer cells in a time- and dose-dependent fashion. The migration and invasion of Y79 cells were inhibited by fisetin in a dose-dependent way. Fisetin inhibited the VEGFR expression in Y79 cells as well as the angiogenesis of a tumor. Therefore, fisetin was found to inhibit angiogenesis through the inhibition of the VEGF/VEGFR signaling pathway and might be used as a candidate drug to inhibit angiogenesis in this cancer [134].

### 3.20. Lymphoma

The inhibition of cell viability and the induction of apoptosis were seen after the fisetin treatment in Raji cells. To recognize the pro-apoptotic action of fisetin on cancer cells, a proteome array of 35 antibodies against apoptosis-associated proteins was applied. It was noticed that there was a decreased protein expression of cIAP-2 after fisetin treatment (30 μM). The downregulation of cIAP-2 by fisetin treatment with the same concentration was established. So, it was examined whether fisetin inhibits mTOR with the same concentration (30 μM), and it was seen that fisetin powerfully inhibited the phosphorylation of mTOR and its downstream targets p70S6K and 4E-BP1 [135].

### 3.21. Thyroid Cancer

Thyroid cancer is a common endocrine neoplasm, with a prevalence of 1.7% of total cancers diagnosed each year in the USA [136]. Fisetin initiated cytotoxic effects as well as the action potential of fisetin on cell proliferation in Human Thyroid TPC 1 Cancer Cells. Fisetin induced apoptosis, which was established via decreased cell viability, improved ROS and altered MMP and cell cycle phases in TPC-1 cells. Additionally, fisetin upregulated the expression of caspase (-3, -8 and -9) expressions and downregulated JAK 1 as well as STAT3 expression in TPC1 cells. Therefore, fisetin induced apoptosis in TPC-1 cells via the initiation of oxidative damage and enhanced caspases expression by downregulating STAT3 and JAK 1 signaling molecules [63] ( Table 2).

## 4. Synergistic Effect of Fisetin with Anticancer Drugs

**Figure 5:**
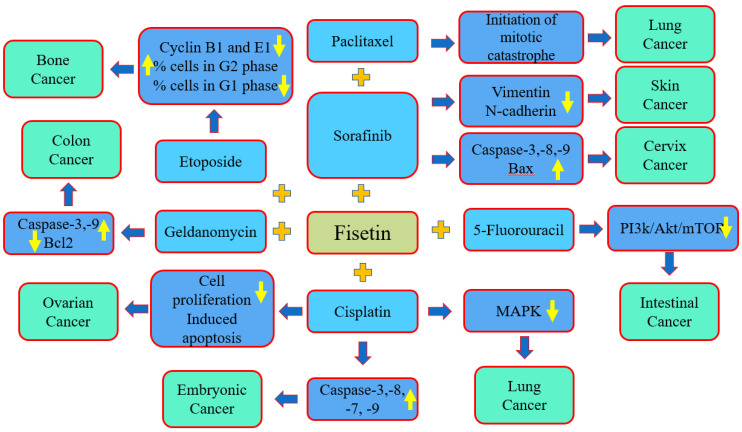
Synergistic role of fisetin with cancer drugs/natural compounds. Up arrow (↑) indicates upregulation, and downarrow (↓) indicates downregulation. The plus sign (+) sign indicates combination of two compounds.

The co-administration of natural compounds and anti-tumor drugs has been used in many experiments, and the results have recognized a reduction in the adverse effects of anticancer drugs by enhancing and inhibiting various genetic pathways. Previous results indicate that the combination of different anti-cancer agents with several natural compounds, including resveratrol, curcumin and epigallocatechin-3-gallate (EGCG), among others, showed the potential to decrease the resistance of cancer treatment and to accomplish chemoprotective actions [137,138,139]. Moreover, the synergistic role of fisetin with cancer drugs or other natural compounds has been proven (Figure 5, Table 3).

An experiment was performed based on in vivo and in vitro studies to evaluate the anti-cancer potential of fisetin combined with the sorafenib against cervical cancer cells. The results of the study revealed that this combination synergistically promoted the induction of apoptosis in HeLa cells, which is convoyed by a noticeable mitochondrial membrane potential loss. The induction of apoptosis was accomplished by the activation of caspase-3,8 which enhanced the ratio of Bax/Bcl-2 and caused the consequent cleavage of the PARP level, whereas it induced the mitochondrial membrane potential in cervix cancer HeLa cells. Overall, the findings indicate that the combination of fisetin and sorafenib showed good synergistic effects in vitro as well as in vivo compared to either compound used alone against human cervical cancer [125] (Figure 5).

Fisetin is used to enhance the cytotoxicity of cisplatin in human embryonal carcinoma NT2/D1 cells. Fisetin and cisplatin increased cisplatin cytotoxicity in vitro at a dose that was four times lower than that needed by cisplatin monotherapy for the same effects of cytotoxicity. Cisplatin and fisetin, as single compounds, activated caspases-8 and -3 as well as caspases-9 and -7, respectively, while the combination treatment stimulated all four caspases. In an NT2/D1 mouse xenograft model, it was reported that the combination agents were the most effective in decreasing the tumor size [140]. The apoptosis encouraged by cisplatin along with fisetin in a cisplatin-resistant ovarian cancer cell line (A2780) was examined. The combined treatment of fisetin and cisplatin efficiently inhibited the proliferation of A2780 cells, and the fragmentation of chromatin in cells occurred in the combination treatment.

The overall findings indicate that the combined use of cisplatin and fisetin enhanced the apoptosis induction in cisplatin-resistant ovarian cancer cells; thus, the combined use of cisplatin and fisetin can be a promising strategy in the treatment of ovarian cancer [141]. A study was performed to examine whether fisetin was able to enhance the cytotoxicity of cisplatin in cisplatin-resistant NSCLC cells and to determine the probable signaling pathways involved. The results indicated that fisetin efficiently increased the sensitivity of A549-CR cells to cisplatin, which was probably mediated via the inhibition of the abnormal activity of MAPK signaling pathways [142]. Fisetin treatment up to 50 µM caused cell cycle G2 phase arrest. Irrespective of the combination, etoposide:fisetin decreased the percentage of cells in the G1 phase and increased the percentage of cells in the G2 phase. Furthermore, mixtures with additional positive ratio combined effects increased the percentage of cells in the S-phase. In comparison to etoposide treatment alone, combinations therapy caused reduced levels of cyclins B1 and E1. Briefly, these findings showed that the combination of fisetin with etoposide has greater anti-proliferative effects in osteosarcoma, linked with the arrest of the cell cycle [131].

Another study based on prostate cancer reported that combination of fisetin increases cabazitaxel and synergistically decrease 22Rν1, PC-3M-luc-6, and C4-2 cell viability and metastatic properties with negligeable adverse effects on normal prostate epithelial cells. Also, the combination of fisetin with cabazitaxel was associated with inhibition of proliferation and enhancement of apoptosis. Combination treatment caused in the inhibition of tumor growth, invasion, as well as metastasis when examined in two in vivo xenograft mouse models [143].

An important study was conducted to evaluate whether fisetin caused the anti-invasive as well as anti-metastatic potential of sorafenib in BRAF-mutated melanoma. It was noticed that the treatment of sorafenib and fisetin together efficiently decreased the migration as well as the invasion of BRAF-mutated melanoma cells. The combination treatment powerfully prevented EMT, as noticed by a reduction in vimentin, N-cadherin as well as fibronectin and an enhancement in E-cadherin both in xenograft tumors and in in vitro conditions. Additionally, combination therapy excellently inhibited Twist1, Snail1, Slug and ZEB1 protein expression compared to individual therapy. The expression of MMP-2, 9 in xenograft tumors was decreased in the combination treatment as compared to separate compounds [144]. A study was conducted to check whether fisetin, alone or in combination with 5-fluorouracil, affected tumorigenesis in the mammalian intestine. The roles of fisetin, 5-fluorouracil or their combination in *PIK3CA* wild-type and *PIK3CA*-mutant colon cancer cells were determined. The treatment of *PIK3CA*-mutant cells with fisetin and 5-fluorouracil decreased the expression of PI3K, the phosphorylation of AKT, mTOR and its target proteins. Additionally, the combination of fisetin and 5-fluorouracil also decreased the total number of intestinal tumors [145].

The cytotoxic effect of the flavonoid fisetin on human colon cancer cells (COLO205) in the presence and absence of geldanamycin (HSP90 inhibitors) and radicicol was evaluated. Compared to the treatment of colon cancer cells (COLO205) with fisetin alone, geldanamycin and radicicol significantly improved the fisetin-induced cytotoxicity, produced a greater density of DNA ladder formation and increased the expression of cleaved caspase-3 and the PAPR protein. Geldanamycin and radicicol decreased the MMPs with the induction of caspase-9 protein cleavage in fisetin-treated cancer cells. Enhanced caspase-3 and 9 activities were noticed in cancer cells treated with fisetin and geldanamycin or fisetin and radicicol. Moreover, a reduction in the p53 protein with increased ubiquitin-tagged proteins was seen in colon cancer cells treated with geldanamycin and fisetin or geldanamycin and radicicol [146]. A synergistic effect of fisetin with paclitaxel has been noticed in lung cancer cells (A549). This synergism was indicated by the initiation of a mitotic catastrophe.

Furthermore, it was demonstrated that the synergism between fisetin and paclitaxel was cell-line-specific and that fisetin synergizes with arsenic trioxide but not with methotrexate and mitoxantrone in the lung cancer cells [105]. The SaOS-2 OS cell line was treated with fisetin alone and in combination with a common chemotherapy drug, etoposide. In cancer cells, SaOs-2’s viability was decreased by 35% after exposure with 60 µM of fisetin. A drug combination analysis with isobologram revealed that, depending on the combination scheme, fisetin showed synergistic cytotoxic effects with etoposide. After 48 h of treatment, up to 50 µM of fisetin induced an increase in the percentage of cells in the G2/M phase (up to 3.3-fold) and decreased the percentage of cells in the G1/G0 phase (down to 0.37-fold) [147]. (Table 3)

## 5. Pharmacokinetics of Fisetin and Strategies to Improve its Bioavailability

Fisetin is an important flavonoid; its health-promoting effects have been vastly described, but there is a limitation of its effectiveness. The usefulness of this compound is due to its low bioavailability and rapid metabolism. The therapeutic role of fisetin has been limited due to its low oral bioavailability (44.1%) [148] and poor aqueous solubility (10.45 μg/mL) [149]. Experiments were performed to evaluate the pharmacokinetics as well as the metabolism of fisetin in mice to examine the therapeutic potential of fisetin metabolites. Fisetin was given at an effective dosage of 223 mg/kilogram intraperitoneally in mice. The plasma concentration declined biophysically, with a rapid half-life of 0.09 h and a terminal half-life of 3.1 h, and the maximum fisetin concentration was available at 2.5 µg/mL at 15 min. Three metabolites were observed; one of them was a glucuronide of fisetin (M1), and glucuronide (M2) was a glucuronide of a previously unidentified fisetin metabolite (M3). The UV spectrum of M3 was identical to that of fisetin and standard 3,4′,7-trihydroxy-3′-methoxyflavone (geraldol). Furthermore, because M3 co-eluted with standard geraldol in four diverse chromatographic ternary gradient conditions, M3 was then allotted to geraldol. Interestingly, this metabolite was revealed to attain higher concentrations than fisetin in Lewis lung tumors. It was compared with the cytotoxic and antiangiogenic activities of fisetin and geraldol in vitro, and it was observed that the latter was additionally cytotoxic compared to the native compound in the direction of tumor cells and that it might inhibit endothelial cell migration as well as proliferation. Overall, these outcomes suggest that fisetin metabolism plays a significant role in its in vivo anticancer potential [150].

The metabolism and pharmacokinetics of fisetin, 5-hydroxyflavone (5-OH-flavone) and 7-hydroxyflavone (7-OH-flavone) in male Sprague-Dawley rats were examined. After being intravenously given fisetin (10 mg/kg of b.w.), this polyphenol declined fast, and fisetin sulfates/glucuronides emerged promptly. Whereas fisetin (50 mg/kg of b.w.) was administered orally, the fisetin parent form was transiently obtainable in serum only through the absorption phase, while fisetin sulfates/glucuronides dominated. The serum metabolites of fisetin showed a less powerful inhibition on 2,2′-azobis(2-amidinopropane hydrochloride) (AAPH)-induced hemolysis than fisetin. The oral administrations of 40 mg/kg of body weight of 5-hydroxyflavone and 7-hydroxyflavone, the glucuronide of 5-OH-flavone and the sulfate/glucuronide of the 7-OH-flavone were observed in the serum, while they were not detected in the parent forms. Finally, fisetin and the 7-OH-flavone were quickly and broadly biotransformed into their sulfate/glucuronides, while the 5-OH-flavone was exclusively metabolized to glucuronide [151]. The hypothesis of this experiment is that fisetin as well as phase II conjugated forms of fisetin may partially undergo biliary excretion. To examine this hypothesis, male rats were included for this study, and their bile ducts were cannulated via polyethylene tubes for bile sampling. The pharmacokinetic outcomes confirmed that the average area-under-the-curve (AUC) ratios (*k* (%) = AUC_conjugate_/AUC_free-form_) of fisetin, its glucuronides and its sulfates were 1:6:21 in plasma and 1:4:75 in bile, respectively. Mainly, the sulfated metabolites were the chief forms that underwent biliary excretion [152].

Innovative polymeric NPs based on PLGA-PEG-COOH, Poly-(ε-caprolactone) as well as encapsulating fisetin were made as appropriate oral controlled release systems. These NPs exhibited a mean diameter of 140–200 nm, and the fisetin loading percentage ranged from 70 to 82%. In vitro release studies demonstrated that these NPs are capable of keeping and preserving the release of fisetin in gastric simulated conditions, as well as regulating the release in the intestinal medium [153].

Furthermore, the ABTS and DPPH scavenging capacity of fisetin, as well as the α-glucosidase inhibition potential, which was around twenty-fold greater than that of commercial acarbose, were reserved during the nano-encapsulation procedure [153]. The bioavailability of fisetin was determined via encapsulating into poly-lactide-co-glycolic acid nanoparticles (PLGA NPs) as a complex of hydroxyl propyl beta cyclodextrin (HPβCD) in order to measure its anti-cancer activity against breast cancer cells. The PFST-HPβCD inclusion complex (FHIC) was prepared, and the results revealed that FHIC-PNP enhanced the anti-cancer activity and apoptosis of fisetin against MCF-7 breast cancer cells and improved its oral bioavailability, as established by the peak plasma concentration increase as well as the total drug absorbed [154]. A 24-fold enhancement in fisetin’s relative bioavailability was noticed in fisetin nanoemulsion when it was given intraperitoneally, as compared to free fisetin. Moreover, the fisetin nanoemulsion showed antitumor activity in Lewis lung carcinoma-bearing mice at lesser doses (36.6 mg/kg) as compared to 223 mg/kg free fisetin [155]. A liposomal formulation was made with DOPC as well as DODA-PEG2000, having a diameter in the nm range of 173.5 ± 2.4 nm, a high fisetin encapsulation (58%) and a high homogeneity. In vivo-based results demonstrated that liposomal fisetin allowed a 47-fold increase in relative bioavailability as compared to free fisetin. The influence of liposomal fisetin on Lewis lung carcinoma tumor growth in mice at low doses (21mg/kg) allowed for a significant delay in the tumor growth (3.3 days) in comparison to free fisetin with a similar dose (1.6 days) [148].

Fisetin NPs were prepared to examine the anti-inflammatory effect in PM 2.5-induced mice. The results showed that astrocytes activation-associated NF-κB signaling as well as oxidative stress contributed to PM 2.5-caused neuroinflammation, which was meaningfully suppressed by increasing the concentration of fisetin NPs treatment. Similarly, fisetin NPs may directly enhance anti-oxidative stress and the anti-inflammatory way to achieve inhibitory effects on NF-κB-related glial cells activation [156]. An important study was conducted to develop and evaluate fisetin-loaded nanocochleates in order to improve their therapeutic efficacy. Nanocochleates established safety and a sustained release of fisetin at the physiological pH. A 1.3-fold enhancement of in vitro anticancer activity towards human breast cancer MCF-7 cells was noticed. Pharmacokinetics studies in mice demonstrated that nanocochleates that were intraperitonially injected displayed a 141-fold greater relative bioavailability. Developed nanocochleates noticeably enhanced the anticancer efficacy, safety and bioavailability of fisetin [149].

## 6. Clinical Trials and Patents on Fisetin

Fisetin is a plant polyphenol that is proven to have anti-oxidant and anti-cancer potential and plays a role in the modulation of various biological processes. A wide range of preclinical studies have shown its role in various diseases including cancer management through the modulation of different cell signaling molecules. Some studies have been conducted to evaluate the therapeutic implications of fisetin for human subjects. However, further clinical trials are needed to be performed in order to explore the fisetin mechanism in disease management including cancer.

An important study based on colorectal cancer was conducted to measure the usefulness of fisetin supplementation regarding the inflammatory status and matrix metalloproteinase levels in colorectal cancer patients. In this double-blind, randomized, placebo-controlled clinical trial, a total of 37 colorectal cancer patients undergoing chemotherapy were instructed to take either 100 mg of fisetin (*n* = 18) or placebo (*n* = 19) for seven successive weeks. The supplementation started one week prior to chemotherapy and was continued until the end of the second chemotherapy cycle. The levels of interleukins including IL -8,-10, high-sensitivity C-reactive protein (CRP) and metalloproteinases 7 and 9 were evaluated in plasma via ELISA, before and after the treatment intervention. The contributors were 55.59 ± 15.46 years old, and 62.16% were male. After the intervention, the plasma levels of IL-8 and CRP decreased significantly in the fisetin treatment group.

Fisetin supplementation suppressed the values of metalloproteinase 7 levels. However, substantial changes were seen only in concentrations of IL-8 in the fisetin group as compared with the placebo group. As per this outcome, fisetin could improve the inflammatory status in colorectal cancer patients, indicating it to be a novel complementary antitumor agent for these patients [34].

To gain the health-promoting potential of fisetin, numerous recent patents have been prepared in different carriers, and their roles in diseases including cancer have been studied. In this review, recent patents and their application in cancer/tumors are described.

In the patent US2021038667A1, a method was devised for making a *Rhus verniciflua* stokes extract with an increased quantity of fisetin. This process involved adding at least one catalyst, selected from the group containing chromium, platinum, silicon, nickel, copper and the oxides of such metals, to a *R. verniciflua* stokes extract. The concentrated *R. verniciflua* stokes extract showed reactions to converting fisetin confined in the *R. verniciflua* stokes into fisetin, a functional health food composition for preventing or ameliorating cancer. The anticancer pharmaceutical composition for preventing the metastasis of cancer with better antioxidant activity and anticancer activity was studied by increasing the content of the chief functional constituents of natural extracts [157].

In the patent CN111700888A by Deguan et al., 2020, the invention reveals an application of fisetin as well as its salt in the preparation of a medicine for fighting radiation damage. It relates to the technical field of medicines and chiefly discloses fisetin, which can be applied to numerous radioactive organ injuries as well as DNA injuries produced by ionizing radiation and can be applied to supplementary medicines for tumor radiotherapy. The fisetin and the salt broadly exist in nature and have clear effects of preventing and treating various radiation damages, so the invention delivers a new method for the application of fisetin, delivers a new raw material for a radiation damage-resistant treatment and can reduce the medicament production cost and facilitate market popularization [158].

In the patent KR20200027344A (Hyung et al., 2020), the invention offers a pharmacological composition for inhibiting or treating uterine myoma and a food composition for stopping or improving uterine myoma, where the compositions comprise fisetin—more precisely, fisetin derived from a *R. verniciflua* stokes extract as a chief constituent. Fisetin conferring to the current invention can be used as a pharmaceutical composition for preventing or treating uterine myoma [159].

## 7. Conclusions

Fisetin is a powerful antioxidant and anti-inflammatory bioactive compound which is found in good concentrations in strawberries, apples, onions, peaches, grapes and persimmons. Previous findings have confirmed that the consumption of theses fruits and vegetables is associated with a lower risk of cancers and is useful in the prevention of diseases through its antioxidant potential.

Fisetin has been established to display anticancer properties through modulating cell signaling molecules. Moreover, fisetin and anti-cancer drugs have been used as combination therapies, and the results have shown anticancer effects with a higher efficacy and a reduction in the adverse effects of anticancer drugs. Several studies based on nanoformulation have confirmed its role in cancer management through increased efficacy and extended direct delivery to tumor cells. More studies need to be performed to sort out the limitations of the bioavailability of fisetin through nanoformulations for the development of an effective dosage. The combination of fisetin with anti-cancer drugs can further be examined. Moreover, more clinical trials should be performed to explore the anti-cancer potential, the mechanism-of-action and its optimum therapeutic dosage.

## Figures and Tables

**Table 1:** Anticancer molecular target of fisetin and the mechanism of action.

Pathways/Gene	Anticancer Mechanism	Refs.
Inflammation	Plasma levels of hs-CRP as well as interlukin-8 decreased, and the fisetin supplement reduced the values of MMP-7 levels. Fisetin improved the inflammatory status in cancer patients.	[34]
Apoptosis	Treatment with fisetin induced a sub-G1 population in a dose-dependent way. Additionally, the cleavage of poly(ADP-ribose) polymerase (PARP)—a substrate of caspase as well as a marker of apoptosis—was also enhanced. Fisetin caused morphological changes, followed by cell membrane blebbing and shrinkage.	[38]
	Fisetin reduced the mitochondrial membrane potential and the proapoptotic members Bak and Bax and activated caspase-3 and PARP.	[39]
	Fisetin downregulated anti-apoptotic genes and upregulated pro-apoptotic genes. The expression of multiple receptors and ligands involved in extrinsic pathways increased.	[40]
	Fisetin inhibited antiapoptotic Bcl-2 family proteins as well as damaged the mitochondrial transmembrane potential.	[41]
Autophagy	Fisetin induced autophagy, upregulated the autophagy marker LC3B and increased the autophagic flux in pancreatic cancer cells.	[45]
	Fisetin treatment induces the development of autophagic vacuoles in oral cancer cells. Fisetin-induced autophagy in cancer cells was observed via numerous autophagy markers.	[39]
Angiogenesis	Fisetin affected the expression of VEGFR, and this effect was in a dose-dependent manner. Consequently, fisetin downregulated the VEGFR expression.	[47]
	Fisetin inhibited capillary-like tube formation, which was linked with a decreased expression of vascular endothelial growth factor and endothelial nitric oxide synthase.	[48]
	Fisetin treatment caused a dose-dependent decrease in Matrigel plug hemoglobin levels and a decrease in tumor angiogenesis.	[49]
Cell cycle	Fisetin treatment played a role in the cell cycle arrest at the G0/G1 phase. The sub-G1 group meaningfully increased after the treatment of fisetin.	[51]
	Fisetin decreased the total viable cells via G0/G1 phase arrest and induced the sub-G_1_ phase.	[52]
	Fisetin as well as hesperetin treatment caused a concentration- and time-dependent inhibition of proliferation and induced G2/M arrest.	[53]
PI3K/AKT/ mTOR	Fisetin showed a potential role in the regulation of cancer via inducing apoptosis and regulated autophagy through AKT/NF-κB/mTOR signaling pathways.	[54]
	Fisetin suppress the growth, invasion and migration of pancreatic cancer cells through reducing the PI3K/AKT/mTOR cascade.	[55]
	p-Akt and p-Akt/Akt, p-PI3K and p-PI3K/PI3K and p-mTOR decreased, upregulated Bax and downregulated Bcl-xL after fisetin treatment.	[57]
STAT3	Fisetin downregulated the JAK 1 and STAT3 expression in cancer cells, and fisetin induced apoptosis.	[63]
Wnt/ beta-catenin	Fisetin treatment reduced the levels of Wnt-target genes including cyclin D1 and MMP7.	[68]
	Increasing doses of fisetin were linked with a decrease in cytosolic β-catenin, with a concomitant reduction in nuclear β-catenin.	[69]
Nuclear factor-κB	Nuclear factor-κB was accumulated in the cytoplasm, and the protein expression of NF-κB in the nuclei was decreased by fisetin treatment.	[51]
	Fisetin inhibited the enhancement of IKK, causing a reduction in the stimulation of the NF-κB signaling pathway.	[71]
Nrf2	Fisetin translocated Nrf2 into the nucleus, and the expression of the downstream HO-1 gene was upregulated via the inhibition of the Nrf2 degradation at the post-transcriptional level.	[75]
	Fisetin-induced HO-1 protein expression was reduced through silencing Nrf2.	[76]
JNK/ERK/AP-1	Fisetin upregulated ZAK expression to induce the Hippo pathway and mediated the activation of JNK/ERK to trigger cell apoptosis in an AP-1-dependent manner	[79]

**Table 2:** Anticancer effects of fisetin in different types of cancer.

Cancer	Outcome of the Study	Refs.
Prostate cancer	Fisetin is a potent HA synthesis inhibitor which increases the abundance of antiangiogenic HMM-HA and could be used for the management of prostate cancer.	[82]
	The treatment of cancer cells with fisetin showed a high expression of acetylated α-tubulin in a dose-dependent way.	[83]
	Fisetin sensitizes the tumor necrosis factor-related apoptosis-inducing ligand-resistant androgen-dependent LNCaP as well as the androgen-independent prostate cancer cells to tumor necrosis factor-related apoptosis-inducing ligand-induced death.	[84]
	Fisetin acts as a dual inhibitor of mTORC1/2 signaling, leading to the induction of autophagic cell death and the inhibition of Cap-dependent translation in cancer cells.	[85]
Kidney cancer	Fisetin induced a sub-G1 population in a dose-dependent fashion and caused cell shrinkage and membrane blebbing. Cancer cells were highly sensitive, compared to normal cells, to fisetin treatment.	[38]
	Fisetin inhibited the migration and invasion of cancer cells via the downregulation of CTSS, metalloproteinase 9 and disintegrin.	[87]
Liver cancer	Fisetin inhibited autophagy via the activation of PI3K/Akt/mTOR and the modulation of the AMPK signaling pathways.	[89]
	Fisetin displayed a suggestively inhibitory role in cancer cell proliferation. Likewise, the death receptor 2 agonist of bromocriptine absolutely blocked cancer cell proliferation in a dose-dependent fashion.	[90]
Colon cancer	Fisetin dose-dependently blocked both DNA synthesis and cell growth and disturbed cell cycle progression.	[91]
	Fisetin treatment efficiently inhibited the cell viability and apoptosis of CPT11-LoVo cells compared to Oxaliplatin and parental LoVo cancer cells. Moreover, apoptosis was encouraged by fisetin treatment, endorsing Caspase-8 and Cytochrome-C expressions.	[92]
	Fisetin caused an increase in the levels of proapoptotic Bim and Bak and induced a reduction in the protein levels of antiapoptotic Bcl-2 and Bcl-xL.	[93]
	Securin reduction sensitizes human colon cancer cells to fisetin-induced apoptosis.	[94]
Gastric cancer	Fisetin substantially decreases G1 phase cyclins and CDKs levels, and the levels of p53 increased.	[95]
	Fisetin treatment with various concentrations suggestively decreased the proliferation rate of SGC7901 cells.	[96]
Pancreatic cancer	Combination treatment with gemcitabine and fisetin inhibited the proliferation of cancer cells and played role in the induction of apoptosis, as indicated by caspase 3/7 activation.	[97]
	PANC-1 cells were cultured with different concentrations of fisetin, and the findings exhibited that fisetin reduced PANC-1 cell viability in a dose- and time-dependent way.	[45]
	The transient downregulation of DR3 by RNA interference meaningfully increased fisetin-induced changes in cell proliferation, cell invasion and apoptosis.	[99]
Bile duct cancer	Fisetin caused the inhibition of the survival of cancer cells. It also encouraged cellular apoptosis additively in combination with gemcitabine.	[100]
Bladder cancer	Fisetin-induced apoptosis in bladder cancer is initiated through the regulation of two associated pathways: the downregulation of the NF-κB pathway and the upregulation of p53 activity.	[101]
	Fisetin inhibited the proliferation of EJ and T24 cells via blocking cell cycle progression in the G0/G1 phase and inducing apoptosis.	[51]
Lung cancer	Fisetin suppressed proliferation, adhesion, migration and invasion. The induction of apoptosis was noticed after fisetin treatment via decreasing the expression of cyclin-D and c-myc.	[103]
	Fisetin showed some protection against paclitaxel-mediated cytotoxicity.	[104]
	Fisetin efficiently enhanced the sensitivity of erlotinib-resistant cancer cells to erlotinib, possibly via inhibiting the abnormal enhancement of AKT as well as MAPK signaling pathways.	[106]
	Fisetin-treated cells demonstrated a dose-dependent prevention of the constituents of the mTOR signaling complex.	[56]
Skin cancer	Mice previously treated with fisetin binary ethosomes gel showed a noticeable decrease in the levels of the pro-inflammatory marker and a lower percentage of tumor incidences.	[109]
	Fisetin inhibited IKK activation, leading to a reduction in the enhancement of the Nuclear Factor-κB signaling pathway.	[71]
	The treatment of fisetin decreases cell proliferation markers and DNA damage, as demonstrated by the increased expression of p21 and p53 proteins.	[111]
Oral cancer	Fisetin induced cell death, G2/M phase arrest and the induction of apoptosis and decreased the level of mitochondria membrane potential.	[112]
	Fisetin induced apoptotic cell death via increased reactive oxygen species and Ca^2+^. Moreover, fisetin played a role in the increase in caspase-8, -9 and -3 activities as well as reduced the mitochondrial membrane potential.	[113]
	Fisetin expressively inhibits tumor cell proliferation and induces apoptosis in oral squamous cell carcinoma.	[114]
	Fisetin played a role in the inhibition of the enhancement of PI3K/AKT-controlled mTOR.	[54]
Leukemia	Fisetin was able to kill THP-1 cells in vivo, resulting in tumor shrinkage in the xenograft mouse model. Death induction in vitro was facilitated by an increase in the nitric, causing the activation of both the extrinsic and the intrinsic apoptotic pathways.	[117]
Breast cancer	Fisetin-induced apoptosis was revoked by z-VAD-fmk, a pan-caspase inhibitor. Additionally, the inhibition of autophagy by using fisetin was revealed as an extra route to speedy anticancer activity in MCF-7 cells.	[118]
	Fisetin suggestively decreased 2-O-tetradecanoylphorbol-13-acetate-caused cell invasion in breast cancer cells, and it was also found to inhibit the activation of the p38 MAPK.	[119]
	Fisetin inhibited breast cancer cells division and also played a role in the induction of apoptosis.	[120]
	Fisetin enhanced tumor cell apoptosis and decreased the growth of 4T1 cell-derived orthotopic breast tumors.	[57]
Ovarian cancer	Fisetin as well as fisetin nanoparticles showed an inhibitory effect on ovarian cancer cells in a dose-dependent way.	[122]
	In a rat model study, the tumor mass and tumor volume were meaningfully reduced under the effect of fisetin. A greater concentration of fisetin caused a substantial increase in Bax and a decrease in Bcl-2.	[123]
	Cell numbers were reduced after fisetin/fisetin micelles treatment in a time- and dose-dependent fashion.	[124]
Cervix cancer	Synergistic apoptosis was induced by the combination of fisetin and sorafenib in cancer cells.	[125]
	Fisetin induced the apoptosis of cervical cancer (HeLa) cells in a dose- and time-dependent fashion.	[126]
	SiHa cells treated with fisetin concentrations of 20 and 40 µM exhibited a decrease in motility of 46.0% and 81.3%, respectively, and similar findings were also seen in CaSki cells, with 62.1% and 90.2% of inhibition.	[127]
Endometrium cancer	Fisetin (20–100 µM) efficiently decreased the viability of KLE and Hec1 A cells and possibly changed the cell population at the G2/M stage.	[128]
Bone cancer	Fisetin suppressed migration, cell mobility and invasion and inhibited MMP-2 activity in U-2 OS cells.	[129]
	Fisetin was related to a reduction in colony formation in U2OS and Saos-2 cells, whereas it was not in MG-63 and MG-63 cells, and Saos-2 cells showed a reduced cell proliferation at concentrations of 40 and 20 µM of fisetin, respectively.	[130]
	The ERK1/2 and PI3K/Akt pathways were inhibited by fisetin, and fisetin enhanced the expressions of p-c-Jun, p-JNK and p-p38. Fisetin caused reactive oxygen species (ROS) generation as well as a reduction in mitochondrial membrane potential.	[131]
Brain cancer	Fisetin could be a possible therapeutic agent to counter human glioma cells based on its ability to activate ERK1/2 and to prevent the expression of ADAM9.	[132]
	Fisetin was found to have cytotoxicity at lower doses in T98G cells as compared to a positive control.	[133]
Retina cancer	Fisetin inhibited the migration and invasion of Y79 cells in a dose-dependent way.	[47]
Cancer of the lymphatic system	Fisetin (30 μM) treatment played role in the decreased protein expression of cIAP-2.	[135]
Thyroid cancer	Fisetin enhanced the expression of the caspase (-3, -8, and -9) and decreased the JAK 1 and STAT3 expression in cancer cells.	[63]

**Table 3:** Synergistic effects of fisetin with different anticancer drugs.

Fisetin + Anticancer Drugs	Cancer	Finding of the Study	Refs.
Fisetin and sorafenib	Cervix cancer	The combination of sorafenib and fisetin synergistically caused the induction of apoptosis. The combined treatment of fisetin and sorafenib was obviously greater than sorafenib treatment alone based on the HeLa xenograft model.	[125]
Fisetin and cisplatin	Embryonal carcinoma	Fisetin enhanced the cytotoxicity of cisplatin. Fisetin and cisplatin increased cisplatin’s cytotoxicity at a dose that was 4 times lower than that needed by cisplatin monotherapy for the same effects of cytotoxicity.	[140]
Fisetin and cisplatin	Ovarian cancer	The combined treatment of Fisetin and cisplatin efficiently inhibits the proliferation of cancer cells, and the fragmentation of chromatin in cells occurred in the combination treatment.	[141]
Fisetin and cisplatin	Lung cancer	Fisetin efficiently increased the sensitivity of cancer cells to cisplatin, probably mediated via the inhibition of the abnormal activation of MAPK signaling pathways.	[142]
Fisetin and etoposide	Bone cancer	Fisetin:etoposide decreased the percentage of cells in the G1-phase and increased the percentage of cells in the G2-phase.	[131]
Fisetin and sorafenib	Skin cancer	The combination treatment (sorafenib + fisetin) more efficiently decreased the migration and invasion of BRAF-mutated melanoma cells.	[144]
Fisetin and 5-fluorouracil	Colon cancer	The treatment of *PIK3CA*-mutant cells with fisetin and 5-fluorouracil decreased the expression of PI3K, the phosphorylation of AKT and mTOR. Additionally, the combination of fisetin and 5-FU also decreased the total number of intestinal tumors.	[145]
Fisetin and geldanamycin	Colon cancer	Compared to fisetin treatment alone, geldanamycin and radicicol meaningfully increased the fisetin-induced cytotoxicity, produced a greater density of DNA ladder formation and enhanced the expression of cleaved caspase-3 as well as the PAPR protein	[146]
Fisetin and paclitaxel	Lung cancer	The switch from the cytoprotective autophagy to the autophagic cell death was concerned in the role of the synergistic action of both of the used compounds.	[105]
Fisetin and etoposide	Bone cancer	Cancer cells viability was decreased by 35% after exposure with fisetin. Fisetin showed synergistic cytotoxic effects with etoposide.	[147]

## Data Availability

Not applicable.

## References

[1] Devi K.P., Rajavel T., Nabavi S.F., Setzer W.N., Ahmadi A., Mansouri K. (2015). Hesperidin: A promising anticancer agent from nature. Ind. Crop. Prod..

[2] Sung H., Ferlay J., Siegel R.L., Laversanne M., Soerjomataram I., Jemal A., Bray F. (2021). Global Cancer Statistics 2020: GLOBOCAN Estimates of Incidence and Mortality Worldwide for 36 Cancers in 185 Countries. CA Cancer J. Clin..

[3] Siegel R.L., Miller K.D., Jemal A. (2020). Cancer statistics, 2020. CA Cancer J. Clin..

[4] Tompa D.R., Immanuel A., Srikanth S., Kadhirvel S. (2021). Trends and strategies to combat viral infections: A review on FDA approved antiviral drugs. Int. J. Biol. Macromol..

[5] Ayaz M., Ullah F., Sadiq A., Ullah F., Ovais M., Ahmed J., Devkota H.P. (2019). Synergistic interactions of phytochemicals with antimicrobial agents: Potential strategy to counteract drug resistance. Chem. Interact..

[6] Ullah F., Ayaz A., Saqib S., Zaman W., Butt M.A., Ullah A. (2019). *Silene conoidea* L.: A Review on its Systematic, Ethnobotany and Phytochemical profile. Plant Sci. Today.

[7] Liskova A., Koklesova L., Samec M., Smejkal K., Samuel S.M., Varghese E., Abotaleb M., Biringer K., Kudela E., Danko J. (2020). Flavonoids in Cancer Metastasis. Cancers.

[8] Almatroodi S.A., Almatroudi A., Alsahli M.A., Khan A.A., Rahmani A.H. (2020). Thymoquinone, an Active Compound of Nigella sativa: Role in Prevention and Treatment of Cancer. Curr. Pharm. Biotechnol..

[9] Almatroodi S.A., Almatroudi A., Khan A.A., Alhumaydhi F.A., Alsahli M.A., Rahmani A.H. (2020). Potential Therapeutic Targets of Epigallocatechin Gallate (EGCG), the Most Abundant Catechin in Green Tea, and its Role in the Therapy of Various Types of Cancer. Molecules.

[10] Syed M.A., Rahmani A.H. (2021). Potential Therapeutic Targets of Curcumin, Most Abundant Active Compound of Turmeric Spice: Role in the Management of Various Types of Cancer. Recent Patents Anti-Cancer Drug Discov..

[11] Rahmani A.H., Almatroudi A., Khan A.A., Babiker A.Y., Alanezi M., Allemailem K.S. (2022). The Multifaceted Role of Baicalein in Cancer Management through Modulation of Cell Signalling Pathways. Molecules.

[12] Hashemzaei M., Delarami Far A., Yari A., Heravi R.E., Tabrizian K., Taghdisi S.M., Sadegh S.E., Tsarouhas K., Kouretas D., Tzanakakis G. (2017). Anticancer and apoptosis-inducing effects of quercetin in vitro and in vivo. Oncol. Rep..

[13] Almatroodi S.A., Alsahli M.A., Rahmani A.H. (2022). Berberine: An Important Emphasis on Its Anticancer Effects through Modulation of Various Cell Signaling Pathways. Molecules.

[14] Rahmani A.H., Alsahli M.A., Almatroudi A., Almogbel M.A., Khan A.A., Anwar S., Almatroodi S.A. (2022). The Potential Role of Apigenin in Cancer Prevention and Treatment. Molecules.

[15] Almatroudi A., Alsahli M.A., Alrumaihi F., Allemailem K.S., Rahmani A.H. (2019). Ginger: A Novel Strategy to Battle Cancer through Modulating Cell Signalling Pathways: A Review. Curr. Pharm. Biotechnol..

[16] Kubina R., Iriti M., Kabała-Dzik A. (2021). Anticancer Potential of Selected Flavonols: Fisetin, Kaempferol, and Quercetin on Head and Neck Cancers. Nutrients.

[17] Kimira M., Arai Y., Shimoi K., Watanabe S. (1998). Japanese intake of flavonoids and isoflavonoids from foods. J. Epidemiol..

[18] Singh S., Singh A.K., Garg G., Rizvi S.I. (2018). Fisetin as a caloric restriction mimetic protects rat brain against aging induced oxidative stress, apoptosis and neurodegeneration. Life Sci..

[19] Zhang L., Wang H., Zhou Y., Zhu Y., Fei M. (2018). Fisetin alleviates oxidative stress after traumatic brain injury via the Nrf2-ARE pathway. Neurochem. Int..

[20] Wu P.-Y., Lyu J.-L., Liu Y.-J., Chien T.-Y., Hsu H.-C., Wen K.-C., Chiang H.-M. (2017). Fisetin Regulates Nrf2 Expression and the Inflammation-Related Signaling Pathway to Prevent UVB-Induced Skin Damage in Hairless Mice. Int. J. Mol. Sci..

[21] Althunibat O.Y., Al Hroob A.M., Abukhalil M.H., Germoush M.O., Bin-Jumah M., Mahmoud A.M. (2019). Fisetin ameliorates oxidative stress, inflammation and apoptosis in diabetic cardiomyopathy. Life Sci..

[22] Khan N., Syed D.N., Ahmad N., Mukhtar H. (2013). Fisetin: A Dietary Antioxidant for Health Promotion. Antioxidants Redox Signal..

[23] Imran M., Saeed F., Gilani S.A., Shariati M.A., Imran A., Afzaal M., Atif M., Tufail T., Anjum F.M. (2021). Fisetin: An anticancer perspective. Food Sci. Nutr..

[24] Maiuri A., O’Hagan H. (2016). Interplay Between Inflammation and Epigenetic Changes in Cancer. Prog. Mol. Biol. Transl. Sci..

[25] Germolec D.R., Shipkowski K.A., Frawley R.P., Evans E. (2018). Markers of Inflammation. Methods Mol. Biol..

[26] Kay J., Thadhani E., Samson L., Engelward B. (2019). Inflammation-induced DNA damage, mutations and cancer. DNA Repair.

[27] Korniluk A., Koper O., Kemona H., Dymicka-Piekarska V. (2017). From inflammation to cancer. Ir. J. Med. Sci..

[28] Hou J., Karin M., Sun B. (2021). Targeting cancer-promoting inflammation-have anti-inflammatory therapies come of age?. Nat. Rev. Clin. Oncol..

[29] Alsahli M.A., Almatroodi S.A., Almatroudi A., Khan A.A., Anwar S., Almutary A.G., Alrumaihi F., Rahmani A.H. (2021). 6-Gingerol, a major ingredient of ginger attenuates Diethylnitrosamine-induced liver injury in rats through the modulation of oxidative stress and anti-inflammatory activity. Mediat. Inflamm..

[30] Alzohairy M.A., Khan A.A., Alsahli M.A., Almatroodi S.A., Rahmani A.H. (2021). Protective Effects of Thymoquinone, Active Compound of Nigella sativa, on Rats with *Benzo(a)pyrene*-Induced Lung Injury through Regulation of Oxidative Stress and Inflammation. Molecules.

[31] Sun Y., Qin H., Zhang H., Feng X., Yang L., Hou D.-X., Chen J. (2021). Fisetin inhibits inflammation and induces autophagy by mediating PI3K/AKT/mTOR signaling in LPS-induced RAW264.7 cells. Food Nutr. Res..

[32] Almatroodi S., Alnuqaydan A., Alsahli M., Khan A., Rahmani A. (2021). Thymoquinone, the Most Prominent Constituent of Nigella Sativa, Attenuates Liver Damage in Streptozotocin-Induced Diabetic Rats via Regulation of Oxidative Stress, Inflammation and Cyclooxygenase-2 Protein Expression. Appl. Sci..

[33] Jiang K., Yang J., Xue G., Dai A., Wu H. (2021). Fisetin Ameliorates the Inflammation and Oxidative Stress in Lipopolysaccharide-Induced Endometritis. J. Inflamm. Res..

[34] Farsad-Naeimi A., Alizadeh M., Esfahani A., Darvish Aminabad E. (2018). Effect of fisetin supplementation on inflammatory factors and matrix metalloproteinase enzymes in colorectal cancer patients. Food Funct..

[35] Arbiser J.L., Bonner M.Y., Gilbert L.C. (2017). Targeting the duality of cancer. NPJ Precis. Oncol..

[36] Xu W., Jing L., Wang Q., Lin C.-C., Chen X., Diao J., Liu Y., Sun X. (2015). Bas-PGAM5L-Drp1 complex is required for intrinsic apoptosis execution. Oncotarget.

[37] Won D.H., Chung S.H., Shin J.A., Hong K.O., Yang I.H., Yun J.W., Cho S.D. (2019). Induction of sestrin 2 is associated with fisetin-mediated apoptosis in human head and neck cancer cell lines. J. Clin. Biochem. Nutr..

[38] Min K.-J., Nam J.-O., Kwon T.K. (2017). Fisetin Induces Apoptosis Through p53-Mediated Up-Regulation of DR5 Expression in Human Renal Carcinoma Caki Cells. Molecules.

[39] Park B.-S., Choi N.-E., Lee J.H., Kang H.-M., Yu S.-B., Kim H.-J., Kang H.-K., Kim I.-R. (2019). Crosstalk between Fisetin-induced Apoptosis and Autophagy in Human Oral Squamous Cell Carcinoma. J. Cancer.

[40] Afroze N., Pramodh S., Shafarin J., Bajbouj K., Hamad M., Sundaram M.K., Haque S., Hussain A. (2022). Fisetin Deters Cell Proliferation, Induces Apoptosis, Alleviates Oxidative Stress and Inflammation in Human Cancer Cells, HeLa. Int. J. Mol. Sci..

[41] Wang K., Hu D.-N., Lin H.-W., Yang W.-E., Hsieh Y.-H., Chien H.-W., Yang S.-F. (2018). Fisetin induces apoptosis through mitochondrial apoptosis pathway in human uveal melanoma cells. Environ. Toxicol..

[42] Zhou S., Zhao L., Kuang M., Zhang B., Liang Z., Yi T., Wei Y., Zhao X. (2012). Autophagy in tumorigenesis and cancer therapy: Dr. Jekyll or Mr. Hyde?. Cancer Lett..

[43] White E. (2012). Deconvoluting the context-dependent role for autophagy in cancer. Nat. Rev. Cancer.

[44] White E., DiPaola R.S. (2009). The Double-Edged Sword of Autophagy Modulation in Cancer. Clin. Cancer Res..

[45] Jia S., Xu X., Zhou S., Chen Y., Ding G., Cao L. (2019). Fisetin induces autophagy in pancreatic cancer cells via endoplasmic reticulum stress- and mitochondrial stress-dependent pathways. Cell Death Dis..

[46] Potente M., Gerhardt H., Carmeliet P. (2011). Basic and Therapeutic Aspects of Angiogenesis. Cell.

[47] Wang L., Chen N., Cheng H. (2020). Fisetin inhibits vascular endothelial growth factor-induced angiogenesis in retinoblastoma cells. Oncol. Lett..

[48] Bhat T.A., Nambiar D., Pal A., Agarwal R., Singh R.P. (2012). Fisetin inhibits various attributes of angiogenesis in vitro and in vivo--implications for angioprevention. Carcinogenesis.

[49] Touil Y.S., Seguin J., Scherman D., Chabot G.G. (2011). Improved antiangiogenic and antitumour activity of the combination of the natural flavonoid fisetin and cyclophosphamide in Lewis lung carcinoma-bearing mice. Cancer Chemother. Pharmacol..

[50] Park J.H., Jang Y.-J., Choi Y.J., Jang J.W., Kim J.-H., Rho Y.-K., Kim I.J., Kim H.-J., Leem M.J., Lee S.-T. (2013). Fisetin Inhibits Matrix Metalloproteinases and Reduces Tumor Cell Invasiveness and Endothelial Cell Tube Formation. Nutr. Cancer.

[51] Li J., Cheng Y., Qu W., Sun Y., Wang Z., Wang H., Tian B. (2011). Fisetin, a dietary flavonoid, induces cell cycle arrest and apoptosis through activation of p53 and inhibition of NF-kappa B pathways in bladder cancer cells. Basic Clin. Pharmacol. Toxicol..

[52] Tsai Y.H., Lin J.J., Ma Y.S., Peng S.F., Huang A.C., Huang Y.P., Fan M.J., Lien J.C., Chung J.G. (2019). Fisetin inhibits cell proliferation through the induction of G0/G1 phase arrest and caspase-3-mediated apoptosis in mouse leukemia cells. Am. J. Chin. Med..

[53] Adan A., Baran Y. (2015). The pleiotropic effects of fisetin and hesperetin on human acute promyelocytic leukemia cells are mediated through apoptosis, cell cycle arrest, and alterations in signaling networks. Tumor Biol..

[54] Zhang X.J., Jia S.S. (2016). Fisetin inhibits laryngeal carcinoma through regulation of AKT/NF-κB/mTOR and ERK1/2 signaling pathways. Biomed Pharm..

[55] Xiao Y., Liu Y., Gao Z., Li X., Weng M., Shi C., Wang C., Sun L. (2021). Fisetin inhibits the proliferation, migration and invasion of pancreatic cancer by targeting PI3K/AKT/mTOR signaling. Aging.

[56] Khan N., Afaq F., Khusro F.H., Mustafa Adhami V., Suh Y., Mukhtar H. (2012). Dual inhibition of phosphatidylinositol 3-kinase/Akt and mammalian target of rapamycin signaling in human nonsmall cell lung cancer cells by a dietary flavonoid fisetin. Int. J. Cancer.

[57] Sun X., Ma X., Li Q., Yang Y., Xu X., Sun J., Yu M., Cao K., Yang L., Yang G. (2018). Anti-cancer effects of fisetin on mammary carcinoma cells via regulation of the PI3K/Akt/mTOR pathway: In vitro and in vivo studies. Int. J. Mol. Med..

[58] Liu L.J., Leung K.H., Chan DS H., Wang Y.T., Ma D.L., Leung C.H. (2014). Identification of a natural product-like STAT3 dimerization inhibitor by structure-based virtual screening. Cell Death Dis..

[59] Zhou J., Qu Z., Sun F., Han L., Li L., Yan S., Stabile L.P., Chen L.F., Siegfried J.M., Xiao G. (2017). Myeloid STAT3 promotes lung tumorigenesis by transforming tumor immunosurveillance into tumor-promoting inflammation. Cancer Immunol. Res..

[60] Song T.L., Nairismägi M.-L., Laurensia Y., Lim J.-Q., Tan J., Li Z.-M., Pang W.-L., Kizhakeyil A., Wijaya G.-C., Huang D. (2018). Oncogenic activation of the STAT3 pathway drives PD-L1 expression in natural killer/T-cell lymphoma. Blood.

[61] Johnson D.E., O’Keefe R.A., Grandis J.R. (2018). Targeting the IL-6/JAK/STAT3 signalling axis in cancer. Nat. Rev. Clin. Oncol..

[62] Yu H., Lee H., Herrmann A., Buettner R., Jove R. (2014). Revisiting STAT3 signalling in cancer: New and unexpected biological functions. Nat. Rev. Cancer.

[63] Liang Y., Kong D., Zhang Y., Li S., Li Y., Ramamoorthy A., Ma J. (2020). Fisetin Inhibits Cell Proliferation and Induces Apoptosis via JAK/STAT3 Signaling Pathways in Human Thyroid TPC 1 Cancer Cells. Biotechnol. Bioprocess Eng..

[64] Pai S.G., Carneiro B.A., Mota J.M., Costa R., Leite C.A., Barroso-Sousa R., Kaplan J.B., Chae Y.K., Giles F.J. (2017). Wnt/beta-catenin pathway: Modulating anticancer immune response. J. Hematol. Oncol..

[65] Cui C., Zhou X., Zhang W., Qu Y., Ke X. (2018). Is β-Catenin a Druggable Target for Cancer Therapy?. Trends Biochem. Sci..

[66] Qin J.-J., Wang W., Li X., Deokar H., Buolamwini J.K., Zhang R. (2018). Inhibiting β-Catenin by β-Carboline-Type MDM2 Inhibitor for Pancreatic Cancer Therapy. Front. Pharmacol..

[67] Cheng X., Xu X., Chen D., Zhao F., Wang W. (2018). Therapeutic potential of targeting the Wnt/β-catenin signaling pathway in colorectal cancer. Biomed. Pharmacother..

[68] Suh Y., Afaq F., Johnson J.J., Mukhtar H. (2009). A plant flavonoid fisetin induces apoptosis in colon cancer cells by inhibition of COX2 and Wnt/EGFR/NF-kappaB-signaling pathways. Carcinogenesis.

[69] Syed D.N., Afaq F., Maddodi N., Johnson J.J., Sarfaraz S., Ahmad A., Setaluri V., Mukhtar H. (2011). Inhibition of Human Melanoma Cell Growth by the Dietary Flavonoid Fisetin Is Associated with Disruption of Wnt/β-Catenin Signaling and Decreased Mitf Levels. J. Investig. Dermatol..

[70] Kansanen E., Kuosmanen S.M., Leinonen H., Levonen A. (2013). The Keap1-Nrf2pathway: Mechanisms of activation and dysregulation in cancer. Redox Biol..

[71] Pal H.C., Sharma S., Strickland L.R., Katiyar S.K., Ballestas M.E., Athar M., Elmets C.A., Afaq F. (2014). Fisetin Inhibits Human Melanoma Cell Invasion through Promotion of Mesenchymal to Epithelial Transition and by Targeting MAPK and NFκB Signaling Pathways. PLoS ONE.

[72] Menegon S., Columbano A., Giordano S. (2016). The Dual Roles of NRF2 in Cancer. Trends Mol. Med..

[73] Xiang M., Namani A., Wu S., Wang X. (2014). Nrf2: Bane or blessing in cancer?. J. Cancer Res. Clin. Oncol..

[74] Bai X., Chen Y., Hou X., Huang M., Jin J. (2016). Emerging role of NRF2 in chemoresistance by regulating drug-metabolizing enzymes and efflux transporters. Drug Metab. Rev..

[75] Zhang H., Zheng W., Feng X., Yang F., Qin H., Wu S., Hou D.-X., Chen J. (2019). Nrf2–ARE Signaling Acts as Master Pathway for the Cellular Antioxidant Activity of Fisetin. Molecules.

[76] Tsai C.-F., Chen J.-H., Chang C.-N., Lu D.-Y., Chang P.-C., Wang S.-L., Yeh W.-L. (2018). Fisetin inhibits cell migration via inducing HO-1 and reducing MMPs expression in breast cancer cell lines. Food Chem. Toxicol..

[77] Trop-Steinberg S., Azar Y. (2017). AP-1 Expression and its Clinical Relevance in Immune Disorders and Cancer. Am. J. Med. Sci..

[78] Kappelmann M., Bosserhoff A., Kuphal S. (2014). AP-1/c-Jun transcription factors: Regulation and function in malignant melanoma. Eur. J. Cell Biol..

[79] Fu C., Chen M., Tseng Y., Chen M., Zhou Z., Yang J., Lin Y., Viswanadha V.P., Wang G., Huang C. (2019). Fisetin activates Hippo pathway and JNK/ERK/AP-1 signaling to inhibit proliferation and induce apoptosis of human osteosarcoma cells via ZAK overexpression. Environ. Toxicol..

[80] Siegel R.L., Miller K.D., Jemal A. (2018). Cancer statistics, 2018. CA Cancer J. Clin..

[81] Dai C., Heemers H., Sharifi N. (2017). Androgen signaling in prostate cancer. Cold Spring Harb. Perspect. Med..

[82] Lall R.K., Syed D.N., Khan M.I., Adhami V.M., Gong Y., Lucey J.A., Mukhtar H. (2016). Dietary flavonoid fisetin increases abundance of high-molecular-mass hyaluronan conferring resistance to prostate oncogenesis. Carcinogenesis.

[83] Mukhtar E., Adhami V.M., Sechi M., Mukhtar H. (2015). Dietary flavonoid fisetin binds to β-tubulin and disrupts microtubule dynamics in prostate cancer cells. Cancer Lett..

[84] Szliszka E., Helewski K.J., Mizgala E., Krol W. (2011). The dietary flavonol fisetin enhances the apoptosis-inducing potential of TRAIL in prostate cancer cells. Int. J. Oncol..

[85] Suh Y., Afaq F., Khan N., Johnson J.J., Khusro F.H., Mukhtar H. (2010). Fisetin induces autophagic cell death through suppression of mTOR signaling pathway in prostate cancer cells. Carcinog..

[86] Escudier B., Porta C., Schmidinger M., Rioux-Leclercq N., Bex A., Khoo V., Grünwald V., Gillessen S., Horwich A., ESMO Guidelines Committee (2019). Renal cell carcinoma: ESMO Clinical Practice Guidelines for diagnosis, treatment and follow-up. Ann. Oncol..

[87] Hsieh M.-H., Tsai J.-P., Yang S.-F., Chiou H.-L., Lin C.-L., Chang H.-R. (2019). Fisetin Suppresses the Proliferation and Metastasis of Renal Cell Carcinoma through Upregulation of MEK/ERK-Targeting CTSS and ADAM9. Cells.

[88] Si Y., Liu J., Shen H., Zhang C., Wu Y., Huang Y., Gong Z., Xue J., Liu T. (2019). Fisetin decreases TET1 activity and CCNY/CDK16 promoter 5hmC levels to inhibit the proliferation and invasion of renal cancer stem cell. J. Cell. Mol. Med..

[89] Sundarraj K., Raghunath A., Panneerselvam L., Perumal E. (2021). Fisetin Inhibits Autophagy in HepG2 Cells via PI3K/Akt/mTOR and AMPK Pathway. Nutr. Cancer.

[90] Liu X.-F., Long H.-J., Miao X.-Y., Liu G.-L., Yao H.-L. (2017). Fisetin inhibits liver cancer growth in a mouse model: Relation to dopamine receptor. Oncol. Rep..

[91] Lu X., Jung J.I., Cho H.J., Lim D.Y., Lee H.S., Chun H.S., Kwon D.Y., Park J.H.Y. (2005). Fisetin Inhibits the Activities of Cyclin-Dependent Kinases Leading to Cell Cycle Arrest in HT-29 Human Colon Cancer Cells. J. Nutr..

[92] Jeng L., Velmurugan B.K., Chen M., Hsu H., Ho T., Day C., Lin Y., Padma V.V., Tu C., Huang C. (2018). Fisetin mediated apoptotic cell death in parental and Oxaliplatin/irinotecan resistant colorectal cancer cells in vitro and in vivo. J. Cell. Physiol..

[93] Lim D.Y., Park J.H.Y. (2009). Induction of p53 contributes to apoptosis of HCT-116 human colon cancer cells induced by the dietary compound fisetin. Am. J. Physiol. Liver Physiol..

[94] Yu S.-H., Yang P.-M., Peng C.-W., Yu Y.-C., Chiu S.-J. (2011). Securin depletion sensitizes human colon cancer cells to fisetin-induced apoptosis. Cancer Lett..

[95] Sabarwal A., Agarwal R., Singh R.P. (2017). Fisetin inhibits cellular proliferation and induces mitochondria-dependent apoptosis in human gastric cancer cells. Mol. Carcinog..

[96] Yan W., Chen S., Zhao Y., Ye X. (2018). Fisetin inhibits the proliferation of gastric cancer cells and induces apoptosis through suppression of ERK 1/2 activation. Oncol. Lett..

[97] Kim N., Kang M.-J., Lee S.H., Son J.H., Lee J.E., Paik W.H., Ryu J.K., Kim Y.-T. (2018). Fisetin Enhances the Cytotoxicity of Gemcitabine by Down-regulating ERK-MYC in MiaPaca-2 Human Pancreatic Cancer Cells. Anticancer. Res..

[98] Huang C., Zhou S., Zhang C., Jin Y., Xu G., Zhou L., Ding G., Pang T., Jia S., Cao L. (2022). ZC3H13-mediated N6-methyladenosine modification of PHF10 is impaired by fisetin which inhibits the DNA damage response in pancreatic cancer. Cancer Lett..

[99] Murtaza I., Adhami V.M., Hafeez B.B., Saleem M., Mukhtar H. (2009). Fisetin, a natural flavonoid, targets chemoresistant human pancreatic cancer AsPC-1 cells through DR3-mediated inhibition of NF-kappaB. Int. J. Cancer..

[100] Kim N., Lee S.H., Son J.H., Lee J.M., Kang M.J., Kim B.H., Lee J.S., Ryu J.K., Kim Y.T. (2016). Fisetin Reduces Cell Viability Through Up-Regulation of Phosphorylation of ERK1/2 in Cholangiocarcinoma Cells. Anticancer. Res..

[101] Li J., Qu W., Cheng Y., Sun Y., Jiang Y., Zou T., Wang Z., Xu Y., Zhao H. (2014). The Inhibitory Effect of Intravesical Fisetin against Bladder Cancer by Induction of p53 and Down-Regulation of NF-kappa B Pathways in a Rat Bladder Carcinogenesis Model. Basic Clin. Pharmacol. Toxicol..

[102] Jemal A., Siegel R., Ward E., Hao Y., Xu J., Murray T., Thun M.J. (2008). Cancer statistics, 2008. CA A Cancer J. Clin..

[103] Wang J., Huang S. (2018). Fisetin inhibits the growth and migration in the A549 human lung cancer cell line via the ERK1/2 pathway. Exp. Ther. Med..

[104] Klimaszewska-Wiśniewska A., Hałas-Wiśniewska M., Grzanka A., Grzanka D. (2018). Evaluation of Anti-Metastatic Potential of the Combination of Fisetin with Paclitaxel on A549 Non-Small Cell Lung Cancer Cells. Int. J. Mol. Sci..

[105] Klimaszewska-Wisniewska A., Halas-Wisniewska M., Tadrowski T., Gagat M., Grzanka D., Grzanka A. (2016). Paclitaxel and the dietary flavonoid fisetin: A synergistic combination that induces mitotic catastrophe and autophagic cell death in A549 non-small cell lung cancer cells. Cancer Cell Int..

[106] Zhang L., Huang Y., Zhuo W., Zhu Y., Zhu B., Chen Z. (2016). Fisetin, a dietary phytochemical, overcomes Erlotinib-resistance of lung adenocarcinoma cells through inhibition of MAPK and AKT pathways. Am. J. Transl. Res..

[107] Kang K.A., Piao M.J., Madduma Hewage S.R., Ryu Y.S., Oh M.C., Kwon T.K., Chae S., Hyun J.W. (2016). Fisetin induces apoptosis and endoplasmic reticulum stress in human non-small cell lung cancer through inhibition of the MAPK signaling pathway. Tumour Biol..

[108] Islami F., Sauer A.G., Miller K.D., Fedewa S.A., Minihan A.K., Geller A.C., Lichtenfeld J.L., Jemal A. (2020). Cutaneous Melanomas Attributable to Ultraviolet Radiation Exposure by State. Int. J. Cancer.

[109] Moolakkadath T., Aqil M., Ahad A., Imam S.S., Praveen A., Sultana Y., Mujeeb M., Iqbal Z. (2019). Fisetin loaded binary ethosomes for management of skin cancer by dermal application on UV exposed mice. Int. J. Pharm..

[110] Imtiyaz K., Rahmani A.H., Alsahli M.A., Almatroodi S.A., Alam Rizvi M.M. (2022). Fisetin induces apoptosis in human skin cancer cells through downregulating MTH1. J. Biomol. Struct. Dyn..

[111] Pal H.C., Athar M., Elmets C.A., Afaq F. (2014). Fisetin Inhibits UVB-induced Cutaneous Inflammation and Activation of PI3K/AKT/NFκB Signaling Pathways in SKH-1 Hairless Mice. Photochem. Photobiol..

[112] Su C.-H., Kuo C.-L., Lu K.-W., Yu F.-S., Ma Y.-S., Yang J.-L., Chu Y.-L., Chueh F.-S., Liu K.-C., Chung J.-G. (2017). Fisetin-induced apoptosis of human oral cancer SCC-4 cells through reactive oxygen species production, endoplasmic reticulum stress, caspase-, and mitochondria-dependent signaling pathways. Environ. Toxicol..

[113] Shih Y.L., Hung F.M., Lee C.H., Yeh M.Y., Lee M.H., Lu H.F., Chen Y.L., Liu J.Y., Chung J.G. (2017). Fisetin Induces Apoptosis of HSC3 Human Oral Cancer Cells Through Endoplasmic Reticulum Stress and Dysfunction of Mitochondria-mediated Signaling Pathways. In Vivo.

[114] Li Y.-S., Qin X.-J., Dai W. (2017). Fisetin suppresses malignant proliferation in human oral squamous cell carcinoma through inhibition of Met/Src signaling pathways. Am. J. Transl. Res..

[115] Li R., Liang H.Y., Li M.Y., Lin C.Y., Shi M.J., Zhang X.J. (2014). Interference of fisetin with targets of the nuclear factor-κB signal transduction pathway activated by Epstein-Barr virus encoded latent membrane protein 1. Asian Pac. J. Cancer Prev..

[116] Girardi T., Vicente C., Cools J., De Keersmaecker K. (2017). The genetics and molecular biology of T-ALL. Blood.

[117] Ash D., Subramanian M., Surolia A., Shaha C. (2015). Nitric oxide is the key mediator of death induced by fisetin in human acute monocytic leukemia cells. Am. J. Cancer Res..

[118] Chiu S.-J., Yang P.-M., Tseng H.-H., Peng C.-W., Chen W.-S. (2011). Dietary flavonoid fisetin targets caspase-3-deficient human breast cancer MCF-7 cells by induction of caspase-7-associated apoptosis and inhibition of autophagy. Int. J. Oncol..

[119] Noh E.-M., Park Y.-J., Kim J.-M., Kim M.-S., Kim H.-R., Song H.-K., Hong O.-Y., So H.-S., Yang S.-H., Kim J.-S. (2015). Fisetin regulates TPA-induced breast cell invasion by suppressing matrix metalloproteinase-9 activation via the PKC/ROS/MAPK pathways. Eur. J. Pharmacol..

[120] Smith M.L., Murphy K., Doucette C.D., Greenshields A.L., Hoskin D.W. (2016). The Dietary Flavonoid Fisetin Causes Cell Cycle Arrest, Caspase-Dependent Apoptosis, and Enhanced Cytotoxicity of Chemotherapeutic Drugs in Triple-Negative Breast Cancer Cells. J. Cell. Biochem..

[121] Guo G., Zhang W., Dang M., Yan M., Chen Z. (2019). Fisetin induces apoptosis in breast cancer MDA-MB-453 cells through degradation of HER2/neu and via the PI3K/Akt pathway. J. Biochem. Mol. Toxicol..

[122] Guo T., Dong X., Shi G. (2018). In vitro and in vivo Antitumor Effects of Fisetin and Fisetin Nanopartical on Ovarian Cancer. J. Sichuan Univ. Med. Sci. Ed..

[123] Meng Y.-B., Xiao C., Chen X.-L., Bai P., Yao Y., Wang H., Xiao X. (2016). The Antitumor Effects of Fisetin on Ovarian Cancer in vitro and in vivo. J. Sichuan Univ. Med. Sci. Ed..

[124] Xiao X., Zou J., Fang Y., Meng Y., Xiao C., Fu J., Liu S., Bai P., Yao Y. (2018). Fisetin and polymeric micelles encapsulating fisetin exhibit potent cytotoxic effects towards ovarian cancer cells. BMC Complement. Altern. Med..

[125] Lin M.T., Lin C.L., Lin T.Y., Cheng C.W., Yang S.F., Lin C.L., Wu C.C., Hsieh Y.H., Tsai J.P. (2016). Synergistic effect of fisetin combined with sorafenib in human cervical cancer HeLa cells through activation of death receptor-5 mediated caspase-8/caspase-3 and the mitochondria-dependent apoptotic pathway. Tumour Biol..

[126] Ying T.H., Yang S.F., Tsai S.J., Hsieh S.C., Huang Y.C., Bau D.T., Hsieh Y.H. (2012). Fisetin induces apoptosis in human cervical cancer HeLa cells through ERK1/2-mediated activation of caspase-8-/caspase-3-dependent pathway. Arch Toxicol..

[127] Chou R.H., Hsieh S.C., Yu Y.L., Huang M.H., Huang Y.C., Hsieh Y.H. (2013). Fisetin inhibits migration and invasion of human cervical cancer cells by down-regulating urokinase plasminogen activator expression through suppressing the p38 MAPK-dependent NF-κB signaling pathway. PLoS ONE..

[128] Wang Z., Liu W., Wang S., Wei Z. (2015). Fisetin induces G2/M phase cell cycle arrest by inactivating cdc25C-cdc2 via ATM-Chk1/2 activation in human endometrial cancer cells. Bangladesh. J. Pharmacol..

[129] Chen J.K., Peng S.F., Lai K.C., Liu H.C., Huang Y.P., Lin C.C., Huang A.C., Chueh F.S., Chung J.G. (2019). Fistein Suppresses Human Osteosarcoma U-2 OS Cell Migration and Invasion via Affecting FAK, uPA and NF-ĸB Signaling Pathway In Vitro. In Vivo.

[130] Li J.-M., Li W.-Y., Huang M.-Y., Zhang X.-Q. (2015). Fisetin, a dietary flavonoid induces apoptosis via modulating the MAPK and PI3K/Akt signalling pathways in human osteosarcoma (U-2 OS) cells. Bangladesh J. Pharmacol..

[131] de Oliveira J.M.P.F., Pacheco A.R., Coutinho L., Oliveira H., Pinho S., Almeida L., Fernandes E., Santos C. (2018). Combination of etoposide and fisetin results in anti-cancer efficiency against osteosarcoma cell models. Arch. Toxicol..

[132] Chen C.M., Hsieh Y.H., Hwang J.M., Jan H.J., Hsieh S.C., Lin S.H., Lai C.Y. (2015). Fisetin suppresses ADAM9 expression and inhibits invasion of glioma cancer cells through increased phosphorylation of ERK1/2. Tumour Biol..

[133] Pak F., Oztopcu-Vatan P. (2019). Fisetin effects on cell proliferation and apoptosis in glioma cells. Z. Nat. C J. Biosci..

[134] Kivelä T. (2009). The epidemiological challenge of the most frequent eye cancer: Retinoblastoma, an issue of birth and death. Br. J. Ophthalmol..

[135] Lim J.Y., Lee J.Y., Byun B.J., Kim S.H. (2015). Fisetin targets phosphatidylinositol-3-kinase and induces apoptosis of human B lymphoma Raji cells. Toxicol. Rep..

[136] Siegel R., Ma J., Zou Z., Jemal A. (2014). Cancer statistics, 2014. CA Cancer J Clin..

[137] Shakibaei M., Kraehe P., Popper B., Shayan P., Goel A., Buhrmann C. (2015). Curcumin potentiates antitumor activity of 5-fluorouracil in a 3D alginate tumor microenvironment of colorectal cancer. BMC Cancer.

[138] Buhrmann C., Yazdi M., Popper B., Shayan P., Goel A., Aggarwal B.B., Shakibaei M. (2018). Resveratrol Chemosensitizes TNF-β-Induced Survival of 5-FU-Treated Colorectal Cancer Cells. Nutrients.

[139] Wang X., Jiang P., Wang P., Yang C.S., Wang X., Feng Q. (2015). EGCG Enhances Cisplatin Sensitivity by Regulating Expression of the Copper and Cisplatin Influx Transporter CTR1 in Ovary Cancer. PLoS ONE..

[140] Tripathi R., Samadder T., Gupta S., Surolia A., Shaha C. (2011). Anticancer Activity of a Combination of Cisplatin and Fisetin in Embryonal Carcinoma Cells and Xenograft Tumors. Mol. Cancer Ther..

[141] Jafarzadeh S., Baharara J., Tehranipour M. (2021). Apoptosis Induction with Combined Use of Cisplatin and Fisetin in Cisplatin- Resistant Ovarian Cancer Cells (A2780). Avicenna J. Med. Biotechnol..

[142] Zhuo W., Zhang L., Zhu Y., Zhu B., Chen Z. (2015). Fisetin, a dietary bioflavonoid, reverses acquired Cisplatin-resistance of lung adenocarcinoma cells through MAPK/Survivin/Caspase pathway. Am. J. Transl. Res..

[143] Mukhtar E., Adhami V.M., Siddiqui I.A., Verma A.K., Mukhtar H. (2016). Fisetin Enhances Chemotherapeutic Effect of Cabazitaxel against Human Prostate Cancer Cells. Mol. Cancer Ther..

[144] Pal H.C., Diamond A.C., Strickland L.R., Kappes J.C., Katiyar S.K., Elmets C.A., Athar M., Afaq F. (2016). Fisetin, a dietary flavonoid, augments the anti-invasive and anti-metastatic potential of sorafenib in melanoma. Oncotarget.

[145] Khan N., Jajeh F., Eberhardt E.L., Miller D.D., Albrecht D.M., Van Doorn R., Hruby M.D., Maresh M.E., Clipson L., Mukhtar H. (2019). Fisetin and 5-fluorouracil: Effective combination for PIK3CA-mutant colorectal cancer. Int. J. Cancer.

[146] Wu M.-S., Lien G.-S., Shen S.-C., Yang L.-Y., Chen Y.-C. (2013). HSP90 Inhibitors, Geldanamycin and Radicicol, Enhance Fisetin-Induced Cytotoxicity via Induction of Apoptosis in Human Colonic Cancer Cells. Evidence-Based Complement. Altern. Med..

[147] de Oliveira J.F., Oliveira H., Pinho S., Pimentel F., Almeida L., Van Zoelen E., Santos C. (2014). Cytotoxic and genotoxic activity of fisetin (3, 3′, 4′, 7-tetrahydroxyflavone) in an osteosarcoma in vitro model. Planta Med..

[148] Seguin J., Brullé L., Boyer R., Lu Y.M., Ramos Romano M., Touil Y.S., Scherman D., Bessodes M., Mignet N., Chabot G.G. (2013). Liposomal encapsulation of the natural flavonoid fisetin improves bioavailability and antitumor efficacy. Int. J. Pharm..

[149] Bothiraja C., Yojana B.D., Pawar A.P., Shaikh K.S., Thorat U.H. (2014). Fisetin-loaded nanocochleates: Formulation, characterisation, in vitro anticancer testing, bioavailability and biodistribution study. Expert Opin. Drug Deliv..

[150] Touil Y.S., Auzeil N., Boulinguez F., Saighi H., Regazzetti A., Scherman D., Chabot G.G. (2011). Fisetin disposition and metabolism in mice: Identification of geraldol as an active metabolite. Biochem. Pharmacol..

[151] Shia C.-S., Tsai S.-Y., Kuo S.-C., Hou Y.-C., Chao P.-D.L. (2009). Metabolism and Pharmacokinetics of 3,3′,4′,7-Tetrahydroxyflavone (Fisetin), 5-Hydroxyflavone, and 7-Hydroxyflavone and Antihemolysis Effects of Fisetin and Its Serum Metabolites. J. Agric. Food Chem..

[152] Huang M.-C., Hsueh T.Y., Cheng Y.-Y., Lin L.-C., Tsai T.-H. (2018). Pharmacokinetics and Biliary Excretion of Fisetin in Rats. J. Agric. Food Chem..

[153] Sechi M., Syed D.N., Pala N., Mariani A., Marceddu S., Brunetti A., Mukhtar H., Sanna V. (2016). Nanoencapsulation of dietary flavonoid fisetin: Formulation and in vitro antioxidant and α-glucosidase inhibition activities. Mater. Sci. Eng. C.

[154] Kadari A., Gudem S., Kulhari H., Bhandi M.M., Borkar R.M., Kolapalli V.R.M., Sistla R. (2017). Enhanced oral bioavailability and anticancer efficacy of fisetin by encapsulating as inclusion complex with HPβCD in polymeric nanoparticles. Drug Deliv..

[155] Ragelle H., Crauste-Manciet S., Seguin J., Brossard D., Scherman D., Arnaud P., Chabot G.G. (2012). Nanoemulsion formulation of fisetin improves bioavailability and antitumour activity in mice. Int. J. Pharm..

[156] Xu M.X., Ge C.X., Li Q., Lou D.S., Hu L.F., Sun Y., Xiong M.X., Lai L.L., Zhong S.Y., Yi C. (2020). Fisetin nanoparticles protect against PM2. 5 exposure-induced neuroinflammation by down-regulation of astrocytes activation related NF-κB signaling pathway. J. Funct. Foods.

[157] Park S.J., Kim K.H. (2022). Method for Preparing Rhus Verniciflua Stokes Extract Containing Increased Fisetin Content, and Metastasis-Inhibiting Anticancer Agent Composition Containing Same.

[158] Deguan D.H.L., Dong Y., Wang M., Zhang Y., Wu J. (2020). Application of Fisetin and Salt Thereof to Preparation of Drugs Resisting Radiation Damages, 2020.

[159] Hyung J.M., Jai C.H., Woo L.J. (2020). Composition for Preventing or Treating Uterine Myoma Comprising Fisetin from Rhus Verniciflua Stokes Extract, 2020.

